# The (pro)renin receptor (ATP6ap2) facilitates receptor-mediated endocytosis and lysosomal function in the renal proximal tubule

**DOI:** 10.1007/s00424-021-02598-z

**Published:** 2021-07-06

**Authors:** Marta Figueiredo, Arezoo Daryadel, Gabin Sihn, Dominik N. Müller, Elena Popova, Anthony Rouselle, Genevieve Nguyen, Michael Bader, Carsten A. Wagner

**Affiliations:** 1 Institute of Physiology, University of Zurich, Winterthurerstrasse 190, CH-8057 Zurich, Switzerland; 2 Max Delbrück Center for Molecular Medicine in the Helmholtz Association (MDC), Robert-Rössle-Str. 10, 13125 Berlin, Germany; 3 Experimental and Clinical Research Center, a joint cooperation between the Charité Medical Faculty and the Max Delbrück Center for Molecular Medicine, Berlin, Germany; 4 DZHK (German Centre for Cardiovascular Research), partner site Berlin, Berlin, Germany; 5 College de France, Paris, France; 6 Charite University Medicine Berlin, Berlin, Germany; 7 Institute for Biology, University of Lübeck, Lübeck, Germany

**Keywords:** Proximal tubule, Low molecular weight proteins, Lysosome, H^+^-ATPase, Endocytosis

## Abstract

The ATP6ap2 (Pro)renin receptor protein associates with H^+^-ATPases which regulate organellar, cellular, and systemic acid–base homeostasis. In the kidney, ATP6ap2 colocalizes with H^+^-ATPases in various cell types including the cells of the proximal tubule. There, H^+^-ATPases are involved in receptor-mediated endocytosis of low molecular weight proteins via the megalin/cubilin receptors. To study ATP6ap2 function in the proximal tubule, we used an inducible shRNA *Atp6ap2* knockdown rat model (Kd) and an inducible kidney-specific *Atp6ap2* knockout mouse model. Both animal lines showed higher proteinuria with elevated albumin, vitamin D binding protein, and procathepsin B in urine. Endocytosis of an injected fluid-phase marker (FITC- dextran, 10 kDa) was normal whereas processing of recombinant transferrin, a marker for receptor-mediated endocytosis, to lysosomes was delayed. While megalin and cubilin expression was unchanged, abundance of several subunits of the H^+^-ATPase involved in receptor-mediated endocytosis was reduced. Lysosomal integrity and H^+^-ATPase function are associated with mTOR signaling. In ATP6ap2, KO mice mTOR and phospho-mTOR appeared normal but increased abundance of the LC3-B subunit of the autophagosome was observed suggesting a more generalized impairment of lysosomal function in the absence of ATP6ap2. Hence, our data suggests a role for ATP6ap2 for proximal tubule function in the kidney with a defect in receptor-mediated endocytosis in mice and rats.

**Supplementary Information:**

The online version contains supplementary material available at 10.1007/s00424-021-02598-z.

## Introduction

The (pro)renin receptor ((P)RR) was identified as the receptor for (pro)renin and renin [45, 67]. However, (P)RR expression is found also in organisms lacking a renin-angiotensin system suggesting additional functions [33, 46, 66, 67]. The (P)RR is an essential adaptor protein in the canonical Wnt signaling pathway [15] and participates also in non-canonical Wnt signaling [9, 10, 23–26]. In humans, mutations in (P)RR cause epilepsy and intellectual impairment [58].

Part of the (P)RR has been recognized to be identical to the M8-9 protein, a potential accessory subunit of H^+^-ATPases and (P)RR is thus also known as ATP6ap2 [36]. Additional studies confirmed protein–protein interactions between the (P)RR/ATP6ap2 and H^+^-ATPases [41].

H^+^-ATPases are multi-subunit complexes that mediate the transport of protons driven by hydrolysis of ATP. H^+^-ATPases are composed of a membrane bound V_0_ domain and a cytoplasmic V_1_ domain. Its location is mainly in intracellular organelles like endosomes, lysosomes, golgi apparatus, and secretory vesicles but it can also be found in the plasma membrane in organs like the kidney, epididymis, and bone [8, 71]. H^+^-ATPases are fundamental for acidification of late endosomes and lysosomes allowing membrane trafficking, protein degradation, and protein maturation [19, 37, 71]. ATP6ap2 shares H^+^-ATPase location in the plasma membrane of renal and other cells [1, 17, 30, 59] but also in intracellular organelles [63]. The importance of H^+^-ATPases in endocytosis has been further confirmed in studies in mice lacking the V_0_ domain H^+^-ATPase a4 subunit in the kidney [22, 48]. These animals presented a generalized impairment of proximal tubule functions with glycosuria, phosphaturia, and lysosomal defects. Similarly, humans and mice with mutations or loss of the ClC-5 chloride/proton exchanger, critical for lysosomal acidification and thought to work in tandem with endosomal and lysosomal H^+^-ATPases, show impaired endocytosis and lysosomal function [14, 53, 73]. Also in other organs, H^+^-ATPase-mediated acidification of lysosomes and autophagosomes is critical for their function [20].

ATP6ap2 has been also linked to endocytosis in mouse and drosophila models. Conditional knockout mice for ATP6ap2 in podocytes and cardiomyocytes exhibit accumulation of autophagic vesicles suggesting lysosomal defects [49, 59]. In addition, embryonic fibroblast and podocyte cells in which ATP6ap2 was deleted showed a downregulation of several V_0_ H^+^-ATPase subunits [30]. In drosophila, deletion of ATP6ap2 impaired Wnt signaling by reducing endosomal recycling of frizzled-related receptors, and reduced fluid-phase endocytosis, both functions linked to reduced H^+^-ATPase activity [25].

The kidney proximal tubule is among the most active endocytic tissues reabsorbing a large variety of proteins, small peptides, and peptidomimetic drugs via receptor-mediated and fluid-phase endocytosis [47]. Receptor-mediated endocytosis requires the presence and function of the endocytic receptors megalin and cubilin expressed in the brush border membrane and endocytic apparatus. Moreover, efficient acidification of the endocytic pathway is critical for recycling and processing of proteins, activation of lysosomal enzymes and transporters, and lysosomal signaling via the mammalian target of rapamycin (mTOR) pathway [40, 65, 68]. Several of these processes have been shown to be H^+^-ATPase dependent [34, 37].

To date, the role of ATP6ap2 in the proximal tubule has not been elucidated. Here, we investigated ATP6ap2 function in proximal tubule using two different animal models. A rat model with an inducible shRNA for *Atp6ap2* and an inducible kidney-epithelial cell-specific knockout mouse for ATP6ap2. We report that ATP6ap2 plays a role in cargo reabsorption and processing via receptor-mediated endocytosis, hence, contributing to kidney proximal tubule function.

## Methods

### Animals

#### Generation and breeding of transgenic Atp6ap2 shRNA rats

Transgenic rats were generated as described previously [32]. Briefly, complementary sense and antisense oligonucleotides (r*Atp6ap2*sh3: 5′- TCC CCC TAC AAC CTT GCG TAT AAT TCA AGA GAT TAT ACG CAA GGT TGT AGG TTT TTT A -3′ and r*Atp6ap2*sh4: 5′- CGC GTA AAA AAC CTA CAA CCT TGC GTA TAA TCT CTT GAA TTA TAC GCA AGG TTG TAG G -3′) specifically targeting a sequence in the exon 9 of the rat (P)RR/*Atp6ap2* mRNA were annealed and cloned into the bimodal pINV7 vector (Taconic) using the BbsI and MluI restriction sites.

**Fig. 1:**
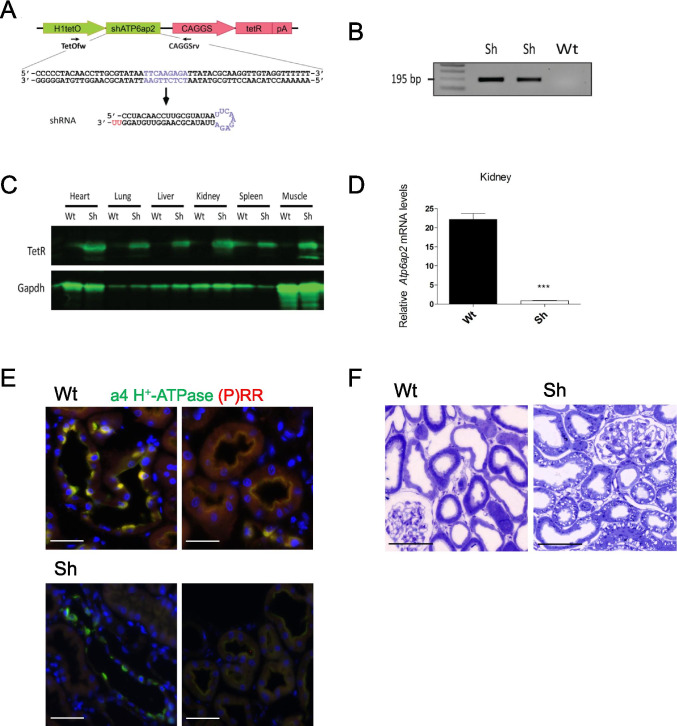
Generation of the *Atp6ap2* shRNA rat model and efficiency of *Atp6ap2*/(P)RR) knock-down. **A** Structure of the transgene construct, pTet-sh, made of two expression cassettes. A first one carries a tetracycline operator (tetO) sequence and expresses an shRNA against *Atp6ap2* under the control of the human H1 promoter. A second cassette consists of a tetracycline repressor (tetR) cDNA followed by a polyadenylation site (pA), and is driven by the CAGGS promoter. Primers TetOfw and CAGGSrv (arrows) were used for genotyping of rats. **B** Genotyping by PCR performed on newborn rat tails with a 195-bp PCR product characteristic of the transgenic animals (Sh). **C** Comparative expression of tetracycline repressor (TetR) protein between transgenic (Sh) and control (Wt) rats, as studied in various tissues by western blot. GAPDH was used as loading control. **D** RT-qPCR analysis of total mRNA of kidneys from rat Wt and Sh demonstrates successful knock-down of (P)RR/*Atp6ap2* mRNA (n = 6/genotype). **E** Immunohistochemistry for the H^+^ATPase a4 subunit (green), ATP6ap2 (red), and DAPI (blue) in kidney sections from Wt and Sh rats. Yellow overlay is seen in the Wt sections due to colocalization of a4 and ATP6ap2 while in sections from Sh rats only green staining for a4 is visible. Scale bar size: 50 µm. **F** Renal morphology of Sh and control animals**.** Semi-thin Sect. (200 nm thick) were stained with Toluidine Blue, scale bar size 100 µm

To generate transgenic rats, a 4 kb DNA fragment containing pTet-shAtp6ap2 (Fig. 1A) was cut out with the PacI and KpnI restriction enzymes, purified from the gel using a Wizard® SV Gel and PCR Clean-Up System (Promega), dissolved at 3 ng/μl with microinjection buffer (8 mM Tris–HCl, pH 7.4, and 0.15 mM EDTA) and microinjected into fertilized oocytes of Sprague–Dawley (SD) rats according to established techniques [54]. Integration of the transgene was detected by PCR in genomic DNA isolated from tail biopsies with the primers TetOfw: 5′ -TGC ATG TCG CTA TGT GTT CT -3′ and CAGGSrv: 5′- TGG CGT TAC TAT GGG AAC AT -3′. Using one positive newborn, the transgenic line Sh*Atp6ap2* was then established on the SD background. Negative littermates were used as wild type (Wt) controls.

Rats were maintained in individually ventilated cages under standardized, pathogen-free conditions (at a temperature of 21 ± 2 °C, humidity of 65 ± 5%, and with an artificial 12-h light/dark cycle) with free access to standard chow (0.25% sodium; SSNIFF) and drinking water ad libitum. All animal care and experiments were performed in accordance with the institutional guidelines of German Federal Law and local authorities of Berlin (LaGeSo).

#### *Doxycycline treatment of rats *in vivo

To induce expression of shRNA against ATP6ap2, animals were treated with 0.5 g/l doxycycline (Sigma) in the drinking water for 8 days. The doxycycline water was freshly prepared each day and kept dark due to the light sensitivity. To confirm the functionality of the system, ATP6ap2 protein and RNA levels were analyzed in organs by western blot and real-time PCR, respectively.

#### Western blotting to confirm shAtp6ap2 rats

Rats were anesthetized with isoflurane followed by exsanguination. Tissues were collected, snap frozen in liquid nitrogen, coarsely ground using a mortar and pestle, and further homogenized in RIPA buffer (Cell Signaling) for protein extraction, using a FastPrep™-24 (MP Biomedicals) according to the manufacturer’s instructions. Total proteins were added to 4 × Roti®-load buffer (Carl Roth) and separated by 12% SDS/PAGE. Proteins were then blotted on a PVDF membrane (Amersham Biosciences), which was subsequently blocked with Odyssey® Blocking Buffer and incubated with the following primary antibodies: mouse anti-TetR (1:8000, MoBiTec) and rabbit anti-GAPDH (1:1000, Cell Signaling). Protein revelation was performed with an Odyssey® Infrared imaging system (LI-COR Biosciences) using IRdye® 800-coupled secondary antibodies (1:10,000, LI-COR Biosciences).

#### Kidney-epithelial cell-specific ATP6ap2 KO mice generation

The generation and genotype of ATP6ap2^flox/y^ has been already described [59]. Heterozygous ATP6ap2 ^flox/x^ females were crossed with males both positive for Pax8-rtTA and Cre [44, 69]. Expression of Cre-recombinase was induced in male ATP6ap2^flox/y,Pax8Cre+^ and ATP6ap2^+/+,Pax8Cre+^ transgenic mice by 1 mg/mL of doxycycline (Sigma) in drinking water containing 2% of Sucrose (Sigma) for 5 days followed by 5 days induction with 0.25 mg/mL doxycycline in 2% sucrose drinking water and 4 days without doxycycline [44]. A second group of ATP6ap2^flox/y,Pax8Cre+^ and ATP6ap2^+/+,Pax8Cre+^ transgenic mice received 2 mg/mL doxycycline for 5 days followed by 5 days of 1 mg/mL doxycycline in water containing 2% sucrose [69]. These groups are accordingly referred to as 1 mg and 2 mg doxycycline. Animals used for experiments were 2- to 3-month-old male mice bred in a C57BL/6 background. All animal studies were performed according to Swiss welfare laws and with approval of the local veterinary authorities.

#### Plasma and urine analysis

Blood was collected from the tail vein of anesthetized rats and vena cava from anesthetized mice. Heparinized blood was centrifuged at 7000 rpm for 7 min, plasma collected, and rapidly frozen. Measurements of blood pH and blood electrolyte concentrations (Na^+^, K^+^, Cl^−^, Ca^2+^) were analyzed using an ABL80 FLEX CO-OX (Radiometer, Copenhagen) and performed on heparinized tail vein blood. Urine was collected under mineral oil for 24 h in metabolic cages (Tecniplast®, Italy) and aliquots were rapidly frozen and stored at − 80 °C until measurement. Urine and plasma laboratory analyses were performed in the Zurich Integrative Rodent Physiology (ZIRP) core facility.

### RNA extraction and RT-qPCR

Harvested organs were snap frozen in liquid nitrogen. Total RNA was extracted using the Qiagen RNeasy Mini Kit (Qiagen, Hombrechtikon, Switzerland). Snap-frozen tissue slices were homogenized in a pestle homogenizer (Potter–Elvehjem type) together with 1 mL precooled RLT-Buffer supplemented with β-mercaptoethanol at a final concentration of 1%. Subsequently, 350 µl of the homogenate was used for RNA preparation carried out according to the manufacturer’s instructions. DNase digestion was performed using the RNase-free DNase Set (Qiagen; Hilden, Germany). Total RNA extractions were analyzed for quality, purity, and concentration using the NanoDrop ND-1000 spectrophotometer (Wilmington, DE, USA). RNA samples were diluted to a final concentration of 100 ng/µl and cDNA was prepared using the TaqMan Reverse Transcriptase Reagent Kit (Applied Biosystems, Roche; Forster City, CA, USA). In brief, in a reaction volume of 40 µl, 100 ng of RNA was used as template and mixed with the following final concentrations of RT buffer (1x), MgCl_2_ (5.5 mM), random hexamers (2.5 µM), dNTP mix (500 µM each), RNase inhibitor (0.4 U/µl), multiscribe reverse transcriptase (1.25 U/µl), and RNAse-free water. Reverse transcription was performed with thermocycling conditions set at 25 °C for 10 min, 48 °C for 30 min, and 95 °C for 5 min on a thermocyler (Biometra, Goettingen, Germany).

Semi-quantitative real-time PCR (RT-qPCR) was performed on the ABI PRISM 7700 Sequence Detection System (Applied Biosystems). Primers for Atp6ap2 were designed using Primer Express software from Applied Biosystems and purchased from Microsynth, Switzerland. Probes were labelled with the reporter dye FAM at the 5′-end and the quencher dye TAMRA at the 3′-end (Microsynth, Balgach, Switzerland).

For *Mus musculus* RT-qPCR, the following pair of primers for *Atp6ap2* was used: forward 5′-GTGCTGGTCGTTCTCCTGTTC- 3′; reverse 5′-GGGATTCGATCTCCTGGTATAG- 3′ together with a probe 5′GTGGCGGGTGCTTTAGGGAATGAAT- 3′ which target only *Atp6ap2* exon 2. For *Rattus norvegicus Atp6ap2* RT-qPCR, we used: forward 5′-TCGGATCCTTGTTGATGCTCTC -3′; reverse 5′- CTCACCAGGGATGTGTCGAA- 3′ primers and 5′- TGGGAATGCAGTGGTAGAGTTGGTGA -3′ as probe. Real-time PCR reactions were performed using TaqMan Universal PCR Master Mix (Applied Biosystems) as described in the manufacturer’s user manual.

### Protein extraction and immunoblotting

Crude membrane and cytosolic proteins were extracted from half a kidney. Briefly, organs were homogenized in 200 μl of ice-cold resuspension buffer (200 mM Mannitol, 80 mM HEPES, 41 mM KOH, pH 7.5 supplemented with 1 tablet/10 mL Complete Mini Protease Inhibitor Cocktail, Roche) using a Polytron homogenizer (0.5 mm diameter at 20,000 rpm for 1 min at 4 °C). The homogenate centrifuged at 2000 rpm for 20 min at 4 °C. The resulting supernatant was further centrifuged at 41,000 rpm for 1 h at 4 °C. Brush border membrane protein preparation was performed according to the Mg^2+^ precipitation procedure [6].

Tissue and urine samples were normalized for protein or creatinine levels, respectively, diluted in Laemmli buffer, heated at 95° for 5 min, and separated by SDS–polyacrylamide gels electrophoresis on 8–12% gels. For immunoblotting, proteins were transferred to polyvinylidene fluoride membranes (Immobilon-P, Millipore, Bedford, MA, USA). After blocking with 5% milk powder in Tris-buffered saline/0.1% Tween-20 for 30 min, blots were incubated with the primary antibodies: rabbit anti-ATP6V0a4 serum (1:1000) [72], rabbit anti-ATP6V0a2 (a kind gift of Dr. X. S. Xie, Dallas, TX, USA) [51, 64], rabbit anti-ATP6V1B2 serum (1:5000 raised against the sequence EFYPRDSAKH, Pineda Berlin, Germany), mouse monoclonal anti-ATP6V1E2 (1:1000, a kind gift of Dr. S. L Gluck, University of California, San Francisco, CA,USA) [4], rabbit polyclonal anti-NaPi-IIa (1:5000) [16], sheep anti-Megalin (1:10.000, a kind gift from Dr. Erik Ilsø Christensen, Aarhus University, Denmark) [42], rabbit anti-cubilin (1:1000, a kind gift from Dr. Erik Ilsø Christensen, Aarhus University, Denmark), rabbit anti-VDBP (1:2500, Dako, A0021), rabbit-human transferrin (1:2000, Dako, A0061), rabbit anti-NHE3 (1:1000, StressMarq,SPC-400D), rabbit anti-(P)RR (1:500, Sigma, HPA003156), mouse anti-Na^+^/K^+^ ATPase α (H-3) (1:5000, Santa Cruz, sc-48345), rabbit anti-mTOR (1:1000, #2972, Cell Signaling Technology, Boston, MA, USA), rabbit anti-phospho-mTOR (1:1000, #2974, Cell Signaling Technology, Boston, MA, USA), and mouse monoclonal anti-β-actin antibody (1:5000, A5441, Sigma), either for 2 h at room temperature or overnight at 4 °C. Membranes were then incubated for 1 h at room temperature with secondary anti-rabbit or anti-mouse antibodies linked to alkaline phosphatase (1:10,000, S373B, S372B, Promega, USA), or anti-sheep antibodies coupled to HRP (1:20,000, Dako, P0163), or anti-mouse HRP (1:20,000, W402B, Promega, USA). The protein signal was detected with the appropriate substrates using the LAS 4000 (Fujifilm) detection system. All images were analyzed using the software Quantity One 4.6.1 (Biorad) to calculate the protein of interest/β-actin ratio.

### Immunofluorescence staining

Mouse and rat kidneys were perfused in situ through the left heart ventricle with a solution containing 10,000 IU Heparin-Na Solution (B. Braun Medical AG, Sempach, Switzerland), 2 mL Lidocain 1% (Streuli Pharma AG, Uznach, Switzerland), 2 mL CaCl_2_, 16%, 2 mL 0.9% NaCl, and 2 mL distilled water. Subsequently, animals were perfused with 3% paraformaldehyde in phosphate-buffered saline (PBS). Fixed kidneys were cryoprotected with 2.3 M sucrose (Sigma), embedded in TissueTec OCT compound 4583 (Sakura Finetek USA, Torrance, CA), and frozen in liquid nitrogen and stored at − 80 °C. Kidney cryosections were cut with 5 µm thickness on a Leica CM3050S cryostat (Leica Microsystems, Bannockburn, IL). Slides were rehydrated in PBS and treated either with 10 mM Tris (Trizma Base, Sigma), pH 10 at 100° for 20 min in a pressure cooker (HistoPro, Millestone) or 10 mM citric acid (Sigma), pH 6.0 at 98 °C for 20 min or 1% sodium dodecyl sulfate (SDS) in PBS. In the case of Lamp1 staining, slides were quenched with 50 mM of NH_4_Cl in PBS, three times for 10 min. Unspecific sites were blocked with a solution containing 1X PBS/5% donkey serum (Sigma)/0.3% Triton X-100 (Sigma) for 20 min at room temperature. Primary antibodies were diluted in 1X PBS/1% BSA/0.3% TritonX-100: goat polyclonal anti-(P)RR/ATP6AP2 (1:100, Novus Biologicals, NB100-1318, used with SDS pretreatment); rabbit polyclonal anti-ATP6IP2 antibody (1:200, ABCAM ab40790, used with microwaving in citric acid), goat polyclonal anti-(P)RR antibody (1:100, R&D Systems, #AF5716, used with SDS pretreatment), rabbit anti- ATP6V0a4 serum (1:2000) [72], goat anti-megalin (1:600, Santa Cruz, sc-16478), rabbit anti-human transferrin (1:600, Dako, A006), rabbit anti-cubilin (1:700, kindly provided by Dr. Erik Ilsø Christensen, Aarhus University, Denmark), rat anti-Lamp1 (1:1000, 1D4B was deposited to the DSHB by August, J.T. (DSHB Hybridoma Product 1D4B)), anti-rabbit LC3B (1:250, Cell Signaling Technology, # 2775), goat anti-AQP2 (1:400, Santa Cruz), or rabbit anti-AQP2 (1:1000, kindly provided by Dr. J. Loffing, University of Zurich) and kidney sections were incubated with the primary antibodies overnight at 4 °C. Sections were washed twice with hypertonic PBS (18 g/l NaCl/PBS) and once with 1X PBS. Afterwards, sections were incubated with corresponding secondary antibodies (1:1000) (anti-goat Alexa594 and anti-goat Alexa488, anti-rat Alexa 647, anti-rabbit Alexa 488, and anti-rabbit Alexa594 (Invitrogen, Switzerland), phalloidin-Texas Red® (1:100, Molecular Probes), phalloidin-A647 (1:1000, Abcam, ab176759), and DAPI (1:1000, Sigma-Aldrich, Buchs, Switzerland) for 1 h at room temperature. Slides were washed twice with hypertonic PBS and then once with 1X PBS before being mounted with Dako glycergel mounting medium. Semi-thin Sects. (200 nm thickness) were cut with an ultramicrotome (Leica Microsystems Leica EM FCS) and stained with Toluidine Blue O (T3260 Sigma-Aldrich). Semi-thin sections were visualized with slide-scanner Axio Scan.Z1 Zeiss, Objective Plan Apochromat 40x, NA 0.95 air (Zeiss Germany). Immunofluorescence sections were either visualized with a confocal laser scanning microscope (Zeiss LSM 700, Carl Zeiss) or a Leica DFC490 charged coupled device camera attached to a Leica DM 6000 fluorescence microscope (Leica, Wetzlar, Germany). The confocal microscope pinhole was set at 1 Airy unit and pixel size at 90 nm and a 40 × /1.3 oil DIC M27 objective was used. Images were processed (overlays) using Adobe Photoshop and ImageJ software (http://rsb.info.nih.gov/ij/).

### Endocytic marker injection and tissue fluorescence measurement

Dextran-FITC 10 kDa (D1821, Molecular Probes) was used as fluid-phase endocytosis marker and human recombinant transferrin (T3795, Sigma) was used as receptor-mediator endocytosis marker [47]. Dextran-FITC and human transferrin were diluted in the same isotonic saline solution (6.25 mg/mL for dextran-FITC and 50 mg/mL for transferrin) and injected into the tail vein. Cytoplasmic fractions for kidney protein extraction were used for measuring FITC in the tissue with Nanodrop 3300 Fluorospectrometer (ThermoScientific). Samples were normalized for protein content and blanked with a sample from an animal that was not injected.

### Statistical analysis

Statistical significances were calculated by Student’s t-test. P < 0.05 was considered significant. Results are presented as mean ± SEM.

## Results

### Knock-down of Atp6ap2 in rats

Transgenic rats (Sh rats) expressing an shRNA against *Atp6ap2* were generated by inserting a transgene under the control of tetracycline repressor protein (TetR) allowing the controlled activation of the shRNA by doxycycline treatment (Fig. 1A). Integration of the transgene and expression of the TetR mRNA and protein was confirmed by PCR (Fig. 1B) and immunoblotting (Fig. 1C). We could detect TetR protein in all organs tested. Expression of the *Atp6ap2* protein and mRNA were studied in more detail in the kidney from animals 10 days after doxycycline treatment. RT-qPCR for *Atp6ap2* mRNA in the whole kidney showed a decrease by approx. 90% of the transcript in Sh rats when compared with control rats (Wt) (Fig. 1D). Similarly, immunohistochemistry on kidney sections stained for the ATP6ap2 and the H^+^-ATPase subunit a4 (Fig. 1E) demonstrated greatly reduced or absent ATP6ap2-related staining in proximal tubule and intercalated cells. H^+^-ATPase subunit a4 staining was used as a positive control, since both proteins share the same localization in the kidney [17]. Thus, *Atp6ap2* shRNA rats present with an efficient knock-down of *Atp6ap2* mRNA and protein in the kidney.

At the level of light microscopy, similar overall renal morphology was seen in Wt and Sh rats (Fig. 1F). However, in Sh rat kidneys, we could observe some areas containing vacuoles, suggesting degeneration of some nephrons. Such areas were less seen in Wt kidneys. The gross morphology of glomeruli was undistinguishable between Wt and Sh rats unlike in mice with podocyte-specific deletion of the ATP6ap2 [49, 59].

Body weight, food and water intake, was not different in Wt and Sh rats 10 days after doxycycline treatment. Urine and blood analysis revealed normal electrolytes and pH. BUN and blood phosphate levels were elevated in Sh rats whereas creatinine clearance decreased consistent with a mild impairment of renal function. In urine, sodium levels were lower while phosphate and creatinine excretion was increased in Sh rats (Table 1).

**Table 1:** Metabolic and urine parameters from Wt and Sh rats. Water and food intake and urine volume were monitored in metabolic cages under baseline conditions. After adaptation, 24 h urine and blood were collected for analysis. Statistical analysis was performed using Student’s *t*-test (n = 6/genotype) *p < 0.05, **p < 0.01, ***p < 0.001

Parameters	Wt	Sh
Body weight (g)	303.8 ± 10.7	268.1 ± 18.7
H_2_O intake (g/g BW)	0.13 ± 0.01	0.13 ± 0.01
Food intake (g/g BW)	0.09 ± 0.01	0.09 ± 0.01
Plasma		
pH	7.47 ± 0.01	7.51 ± 0.02
Glucose (mg/dL)	225.8 ± 22.2	206.8 ± 16.6
Na^+^ (mM)	136.5 ± 1.0	136.2 ± 0.3
K^+^ (mM)	4.37 ± 0.15	3.62 ± 0.22
Ionized Ca^2+^ (mM)	0.84 ± 0.14	0.80 ± 0.05
Cl^−^ (mM)	97.8 ± 0.8	95.7 ± 0.6
Phosphate (mg/dL)	8.6 ± 0.4	9.8 ± 0.3 *
Prealbumin (mg/dL)	1.72 ± 0.31	1.53 ± 0.22
Creatinine (mg/dL)	0.30 ± 0.02	0.40 ± 0.01
BUN (mg/dL)	35.2 ± 1.0	48.3 ± 2.6 ***
Urine		
Urine (g/g BW)	0.04 ± 0.01	0.03 ± 0.01
Creatinine (mg/dL)	72.2 ± 10.3	111.9 ± 13.2 *
Creatinine Clearance (mL/min)	1.9 ± 0.2	1.5 ± 0.2
Na^+^ (mM)/Crea (mg/dL)	95.0 ± 8.3	71.2 ± 22.8 **
K^+^ (mM)/Crea (mg/dL)	2.60 ± 0.11	2.73 ± 0.14
Ca^2+^ (mM)/Crea (mg/dL)	0.020 ± 0.003	Not detectable
Mg^2+^ (mg/dL)/Crea (mg/dL)	0.57 ± 0.10	0.32 ± 0.15
Phosphate (mg/dL)/Crea (mg/dL)	0.08 ± 0.08	1.71 ± 0.20 ***
Cl^−^ (mM)/Crea (mg/dL)	2.2 ± 0.1	2.1 ± 0.1

### ATP6ap2 knock-down impairs proximal tubule receptor-mediated endocytosis

**Fig. 2:**
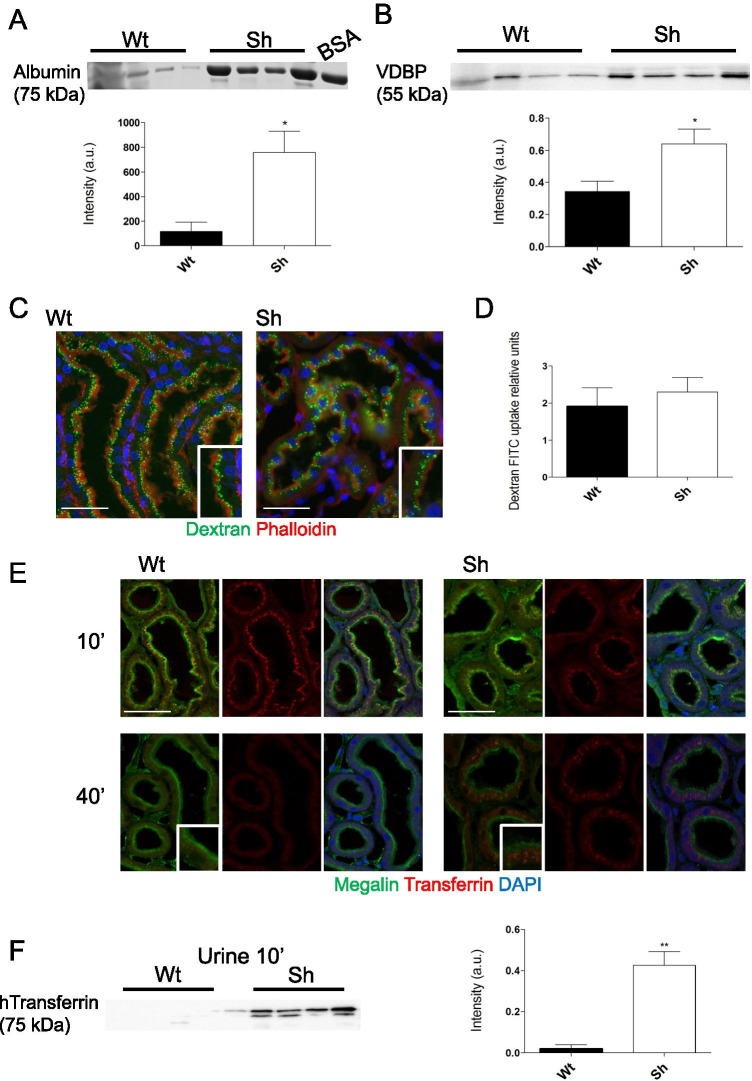
Preserved fluid-phase endocytosis but impaired receptor-mediated endocytosis in (P)RR/ATP6ap2 deficient rats. **A** Albuminuria in Sh rats detected by Coomassie blue staining of SDS-Page gels loaded with urine samples normalized to creatinine (7 mg/mL) BSA (7 mg/mL) was loaded as positive control. Bar graph summarizing data (n = 4 in each animal group). Student’s *t*-test *p < 0.05. **B** Elevated vitamin D binding protein (VDBP) in urine of Sh rats detected by immunoblotting of urine samples normalized to creatinine (7 mg/mL). Bar graph summarizing data from n = 4/genotype. **C** Immunohistochemistry for dextran-FITC (10 kDa, green), DAPI (blue), and actin/phalloidin (red) in kidney slices from Wt and shRNA rat 10 min after injection (scale bar size 50 µm). **D** Quantification of dextran-FITC in the cytoplasmic fraction of the control and Sh rats kidney homogenate. FITC-dextran was normalized to total protein content. **E** Immunohistochemistry for human transferrin (red), megalin (green), and DAPI (blue) in Wt and Sh rats kidney 10 min and 40 min after injection showed strong residual staining of transferrin after 40 min in Sh rat kidneys (see insert) (scale bar size 50 µm). **F** Western blotting for human transferrin in Wt and Sh rat urine 10 min after injection. Urine samples were normalized to creatinine (7 mg/mL). Bar graph summarizing data from n = 4/genotype. Statistical analyses were performed using Student’s *t*-test **p < 0.01

Sh rats exhibited mild albuminuria and low molecular weight proteinuria as indicated by elevated vitamin D binding protein (VDBP) in urine (Fig. 2A,B). To further determine which endocytic pathway may be impaired, we coinjected in both animal groups a marker for fluid endocytosis (dextran-FITC, 10 kDa) and a marker for receptor-mediated endocytosis (human transferrin) and fixed kidneys for immunohistochemistry 10 and 40 min after injection. Dextran-FITC was co-stained with phalloidin (marker for the brush border membrane) to allow subcellular determination of dextran localization. By fluorescence microscopy, no differences were observed in FITC intensity or subcellular localization between animal groups at 10 min. In addition, the relative amount of FITC in kidney tissue at 10 min was similar between animal groups (Fig. 2C,D). Dextran-FITC at 40 min was also analyzed (Supplementary Fig.1) and staining intensity was weaker than at 10 min but showed similar subcellular localization in both genotypes. Thus, fluid-phase endocytosis was not affected by (P)RR/AT6ap2 knock-down.

In contrast, endocytosis of human transferrin was altered in Sh rats compared to Wt animals. We used antibodies recognizing human but not endogenous rat or mouse transferrin and detected a weaker transferrin-related staining in kidneys from Sh rats 10 min after injection whereas 40 min after injection more pronounced staining was found in Sh rat kidneys than in Wt kidneys (Fig. 2E). At 10 min, transferrin was localized in the subapical region and presented as a punctuated pattern as expected [13]. Immunoblotting for human transferrin in urine samples collected 10 min after transferrin injection showed abundant transferrin in urine from Sh rats but very low levels in Wt urine suggesting reduced uptake (Fig. 2F). Forty minutes after injection, in Wt rats, transferrin staining was almost absent while in the Sh rats, transferrin staining accumulated in the perinuclear region (Fig. 2E).

**Fig. 3:**
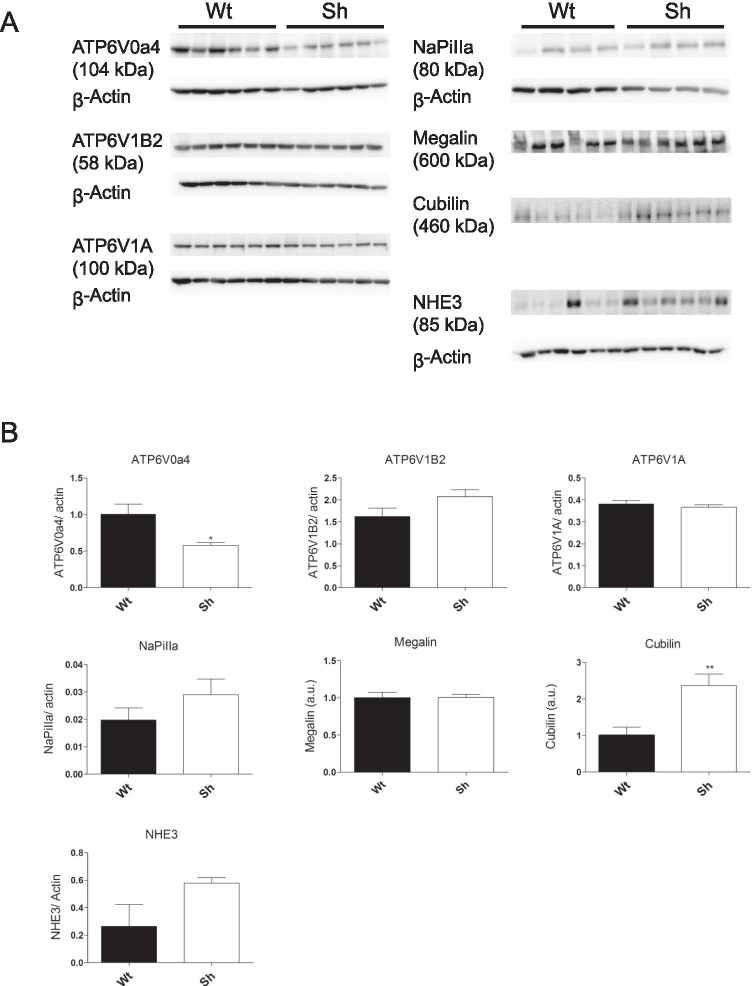
Altered expression of proteins involved in receptor-mediated endocytosis. **A** Brush border membrane preparations of control and Sh rat kidneys were used for blotting for H^+^ATPase a4 (ATP6V0a4), B2 (ATP6V1B2), and A (ATP6V1A) subunits as well as for NaPiIIa, NHE3, megalin, and cubilin. **B** Densitometries were adjusted to β-actin (loading control). Statistical analysis using Student’s *t*-test (n = 6/genotype or n = 4/genotype for NaPiIIa). *p < 0.05 ***p ≤ 0.001

The localization of the two major endocytic receptors megalin and cubilin was similar in both genotypes at light microscopy level (Fig. 2E and supplementary Fig. 2). The relative amount of megalin protein was unchanged whereas cubilin was more abundant in kidneys from Sh rats (Fig. 3).

ATP6ap2 was suggested to be an accessory subunit of H^+^-ATPases [31, 36, 67] therefore a loss of ATP6ap2 could affect H^+^-ATPase regulation and/or function in the endocytic pathway. Immunoblotting showed reduced a4 H^+^-ATPase (ATP6V0a4) subunit whereas B2 (ATP6V1B2) and A (ATP6V1A) subunits were unaltered (Fig. 3).

The abundance of NaPi-IIa, the main phosphate transporter in the proximal tubule, and NHE3 (sodium-proton exchanger 3) is directly or indirectly affected by alterations in the endocytic apparatus of the proximal tubule [2, 3, 18] NaPi-IIa showed no differences in abundance and NHE3 a slight increase in expression, however, not reaching significance due to high interindividual variability (Fig. 3).

### Kidney-epithelial cell-specific ATP6ap2 KO mice

**Fig. 4:**
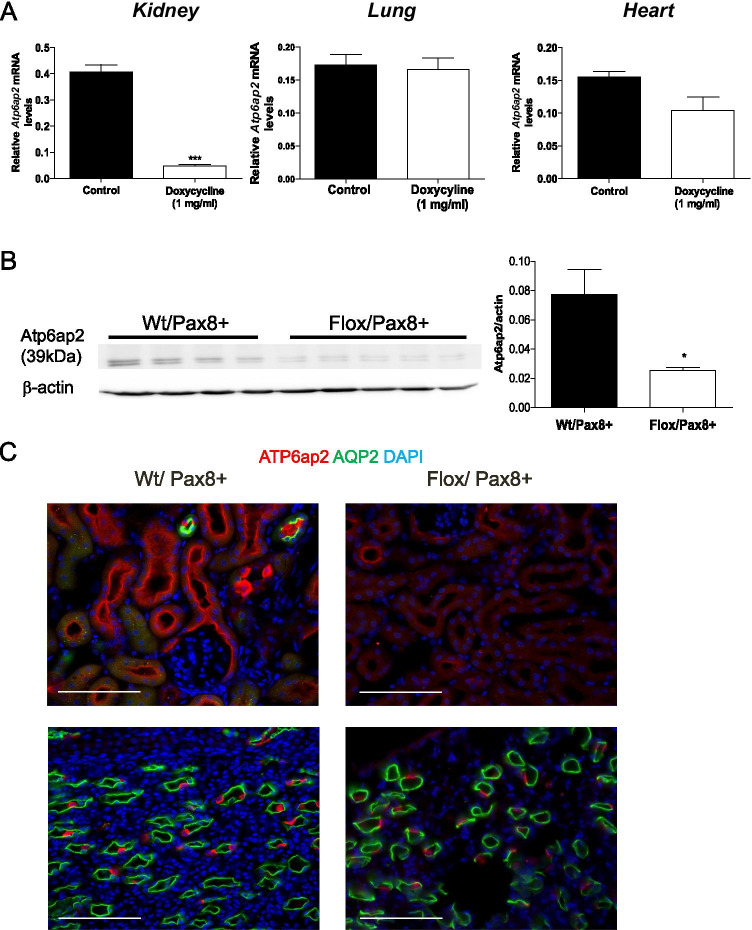
Generation of kidney-epithelial cell-specific ATP6ap2 ablation in mouse. **A** RT-qPCR analysis of total mRNA of kidneys, lungs, and hearts from Wt and Flox/Pax8 + mice treated with 1 mg/mL doxycycline (low dose). *Atp6ap2* mRNA abundance was normalized to *HPRT*. Statistical analysis was performed using Student’s *t*-test (Wt/Pax8 + : n = 4 and Flox/Pax8 + : n = 5) ***p < 0.001. **B** Western blotting for ATP6ap2 in total membrane preparations from the kidney of Wt and Flox/Pax8 + mice and summary of data as bar graph. Statistical analysis was performed using Student’s *t*-test (Wt/Pax8 + : n = 4 and Flox/Pax8 + : n = 5) *p < 0.05. **C** Immunohistochemistry for ATP6ap2 (red), AQP2 (green), and DAPI (blue) in kidney sections from Wt/Pax8 and Flox/Pax8 + mice with proximal tubules (upper panels) and medullary collecting ducts (lower panels). Scale bar size 100 µm

Since *Atp6ap2* shRNA rats present with a full-body knock-down of the ATP6p2 that may impact on overall renal function and that complicates interpretation of data, we decided to examine proximal tubular endocytosis in a kidney-epithelial cell-specific inducible ATP6ap2 knockout (KO) mouse model using the well-described doxycycline-inducible Pax8-Cre system after crossing with floxed ATP6ap2 mice. First, we used a milder induction protocol for Cre activity [44] as usual based on previous experience suggesting that higher doses of doxycycline can also produce effects in wildtype mice and with the intention to downregulate ATP6ap2 mostly in proximal tubules without producing the strong phenotype caused by deletion of ATP6ap2 in the collecting duct [56, 57, 70]. However, to rule out that the milder doxycycline protocol (1 mg/mL induction), was too weak to reveal a proximal tubule role of ATP6ap2, we used in a second and independent series of animals also the conventional doxycycline protocol with 2 mg/mL induction. As detailed below, the findings in both series are qualitatively very similar and we report data from the first series in the main body of this manuscript. The data from the second series are presented in the supplementary files. To verify kidney-specific ATP6ap2 deletion, we performed RT-qPCR for *Atp6ap2* in the kidney, lung, and heart (Fig. 4A and supplementary Fig. 3). *Atp6ap2* was downregulated in both protocols only in kidneys from Flox/Pax8 + mice but not in other organs or kidneys from control Wt/Pax8 + mice. Western blotting using total kidney protein homogenate confirmed reduction of ATP6ap2 protein expression by about 50–75% (Fig. 4B, suppl. Figure 3). Residual mRNA and protein expression may be due to expression in renal cells negative for Pax8 (i.e., podocytes) or incomplete deletion in epithelial cells. Immunohistochemistry using three different antibodies against ATP6ap2 demonstrated almost complete deletion of ATP6ap2 from proximal tubules but widely preserved staining in intercalated cells of the connecting tubule and medullary collecting ducts (Fig. 4C and supplementary Figs. 6 and 7). We noted major differences in specificity of commercial antibodies against ATP6ap2 as shown in Fig. 4 and suppl. Figures 7 and 8.

Flox/Pax8 + mice with the first, lower doxycycline protocol had normal blood acid–base and electrolyte status and no evidence for reduced kidney function unlike rats. Urine analysis showed higher diuresis and mild sodium and potassium losses with reduced urinary calcium excretion consistent with the deletion of the ATP6ap2 mostly from proximal tubules (Table 2). Mice receiving the higher doxycycline dose had mildly elevated plasma creatinine levels but otherwise normal plasma values including BUN. Urine data showed similar urine pH but reduced creatinine clearance in Flox/Pax8 + mice (supplementary table 1). Thus, Flox/Pax8 + with the higher doxycycline protocol showed some signs of renal impairment.

**Table 2:** Metabolic and urine parameters of Wt and Flox/Pax8 + mice. Animals were induced with the low-dose doxycycline protocol (1 mg/mL). Water and food intake were monitored in metabolic cages under baseline conditions. 24 h urine and blood were collected for analysis. Statistical analysis was performed using Student’s *t*-test (n = 8 in both groups) *p < 0.05, **p < 0.01, ***p < 0.001

Parameters	Wt/Pax8 +	Flox/Pax8 +
Body weight (g)	25.2 ± 1.0	23.6 ± 1.8 *
H_2_O intake (g/g BW)	0.19 ± 0.02	0.26 ± 0.01 *
Food intake (g/g BW)	0.16 ± 0.02	0.19 ± 0.01
Plasma		
pH	7.26 ± 0.03	7.27 ± 0.05
Glucose (mg/dL)	250.5 ± 8.9	238.9 ± 15.3
Na^+^ (mM)	144.7 ± 1.1	146.0 ± 1.1
K^+^ (mM)	3.9 ± 0.4	3.2 ± 0.1 *
Ionized Ca^2+^ (mM)	0.96 ± 0.23	1.11 ± 0.05
Cl^−^ (mM)	108.2 ± 1.5	109.2 ± 0.1
Phosphate (mg/dL)	5.9 ± 0.1	6.2 ± 0.3
Prealbumin (mg/dL)	1.76 ± 0.42	1.88 ± 0.15
Creatinine (mg/dL)	0.05 ± 0.01	0.07 ± 0.01
BUN (mg/dL)	40.6 ± 5.9	40.7 ± 3.3
Urine		
Urine (g/g BW)	0.051 ± 0.009	0.083 ± 0.008 *
Creatinine (mg/dL)	63.0 ± 4.2	42.8 ± 2.9 **
Creatinine clearance (μL/min)	109 ± 35	98 ± 23
Na^+^ (mM)/Crea (mg/dL)	1.7 ± 0.2	5.8 ± 1.1 **
K^+^ (mM)/Crea (mg/dL)	6.0 ± 0.3	7.1 ± 0.3*
Ca^2+^ (mM)/Crea (mg/dL)	0.03 ± 0.01	0.01 ± 0.21*
Mg^2+^ (mg/dL)/Crea (mg/dL)	1.65 ± 0.10	1.70 ± 6.25
Phosphate (mg/dL)/Crea (mg/dL)	6.7 ± 0.4	7.1 ± 0.6
Cl^−^ (mM)/Crea (mg/dL)	4.0 ± 0.2	5.0 ± 0.3

### ATP6ap2 deletion in proximal tubule causes a defect in receptor-mediated endocytosis

**Fig. 5:**
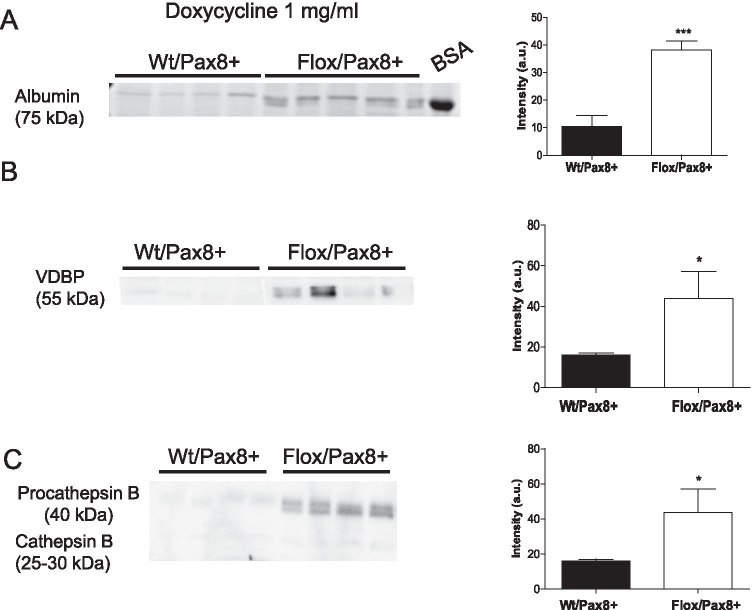
Albuminuria and low molecular weight proteinuria in the absence of the ATP6ap2. Urine samples were normalized to creatinine. Bovine serum albumin (BSA, 7 mg/mL) was loaded as positive control. **A** Albumin was detected by Coomassie blue staining whereas **B** vitamin D binding protein (VDBP) or **C** (Pro)cathepsin B was revealed by immunoblotting. Data were summarized as bar graphs (n = 4/genotype. Student’s *t*-test *p < 0.05, ***p < 0.001

**Fig. 6:**
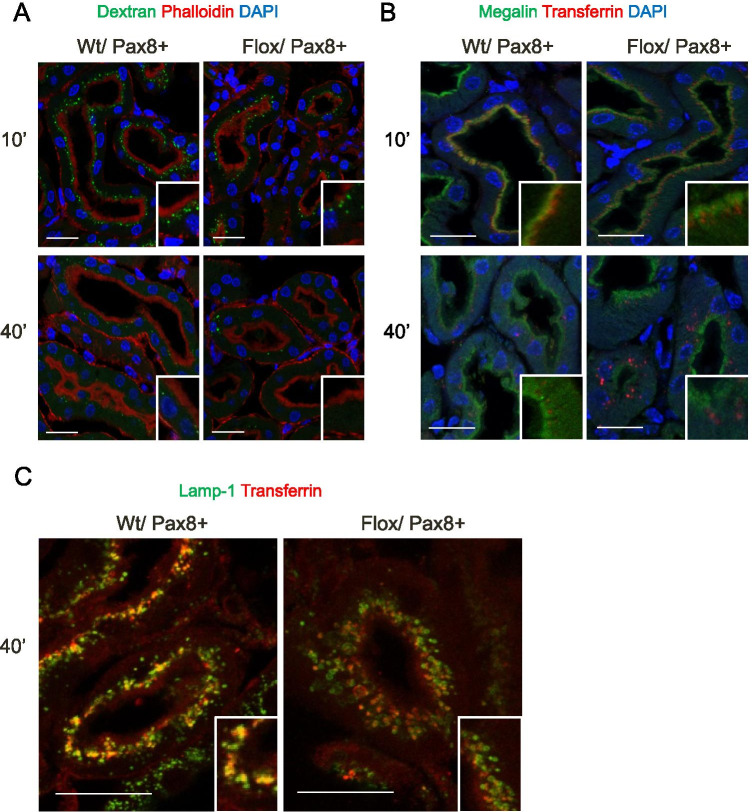
Delayed receptor-mediated endocytosis in ATP6ap2 deleted mice. WT/Pax8 + and Flox/Pax8 + mice were coinjected with dextran-FITC (10 kDa) and human recombinant transferrin. Kidneys were collected 10 and 40 min after injection. **A** Immunohistochemistry for dextran-FITC,10 kDa (green), DAPI (blue), and actin/phalloidin (red) in kidney slices from Wt and Flox/Pax8 + mice 10 and 40 min after injection. **B** Immunohistochemistry for human transferrin (red), DAPI (blue), and megalin (green) in kidneys from Wt and Flox/Pax8 + mice 10 and 40 min after injection. **C** Immunohistochemistry for Lamp-1 (green) and human transferrin (red) 40 min after injection. Scale bar size 50 µm for all pictures

Like the rat model, Flox/Pax8Cre + mice had albuminuria and low molecular weight proteinuria loosing VDBP and procathepsin B with urine (Fig. 5 and supplementary Fig. 4). In the low doxycycline group, coinjection of the endocytic markers dextran-FITC (10 kDa) and human transferrin confirmed that deletion of the ATP6ap2 affects the receptor-mediated endocytosis pathway (Fig. 6). Dextran-FITC localization and intensity of staining was similar between animal groups at two time points (10 and 40 min after injection). Human transferrin staining was similar between genotypes 10 min after injection but showed, like in Sh rats, delayed clearance of transferrin in Flox/Pax8 + mice 40 min after injection with perinuclear accumulation (Fig. 6B). Costaining of human transferrin with Lamp1, a lysosomal marker, in kidneys 40 min after injection (Fig. 6C), revealed that in ATP6ap2-deficient animals human transferrin did not overlap with Lamp-1 stained lysosomes suggesting delayed transferrin processing (Fig. 6C). The same reduced colocalization of recombinant human transferrin and Lamp-1 was seen 40 min after injection in mice receiving the higher dose of doxycycline (supplementary Fig. 5).

**Fig. 7:**
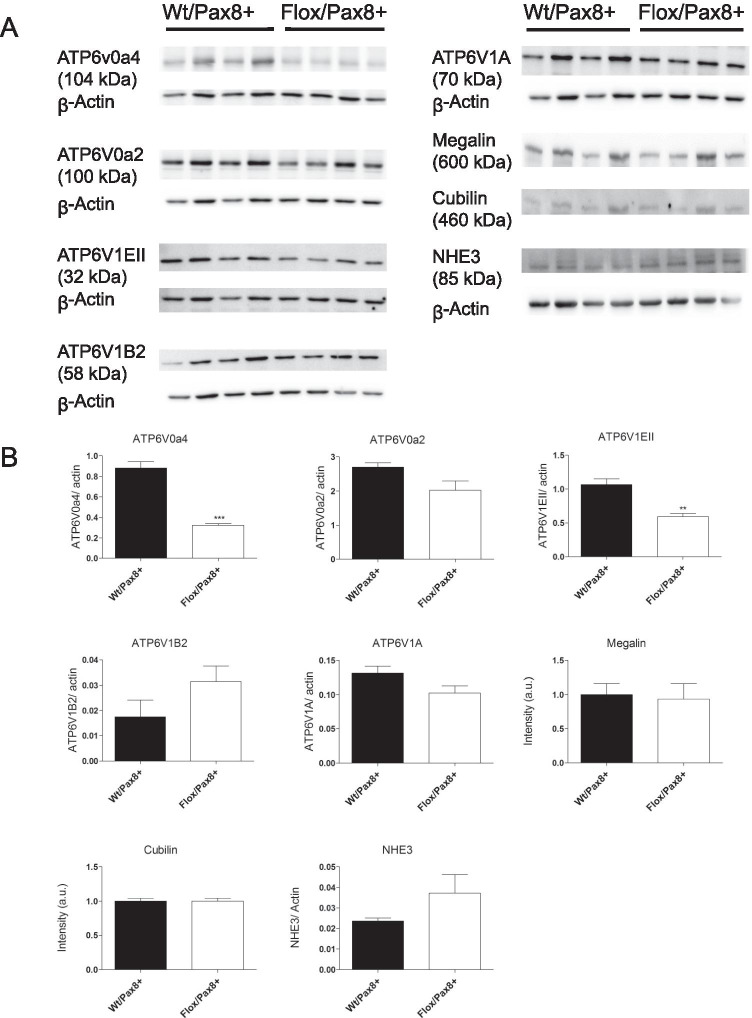
Deletion of the ATP6ap2 alters expression of major proteins in the endocytic pathway. **A** Brush border membrane preparations from kidneys of Wt/Pax8 + and Flox/Pax8 + mice were blotted for the H^+^ATPase a4 (ATP6V0a4), a2 (ATP6V0a2), EII (ATP6V1EII), B2 (ATP6V1B2), and A (ATP6V1A) subunits as well as for NHE3, megalin, and cubilin. **B** Densitometries were normalized to β-actin (loading control). Student’s *t*-test (n = 4 per group), **p ≤ 0.01, ***p ≤ 0.001

Western blotting demonstrated reduced expression of the a4 (ATP6V0a4) and EII (ATP6V1EII) H^+^-ATPase subunits whereas the a2 (ATP6V0a2) and A (ATP6V1A) subunits showed a strong trend to be lowered. ATP6V1B2 protein abundance was unaltered. Megalin, cubilin, and NHE3 abundance was not altered (Fig. 7). Similar results were obtained for the a4 and EII H^+^-ATPase subunits and megalin in the second group of animals (supplementary Fig. 6).

**Fig. 8:**
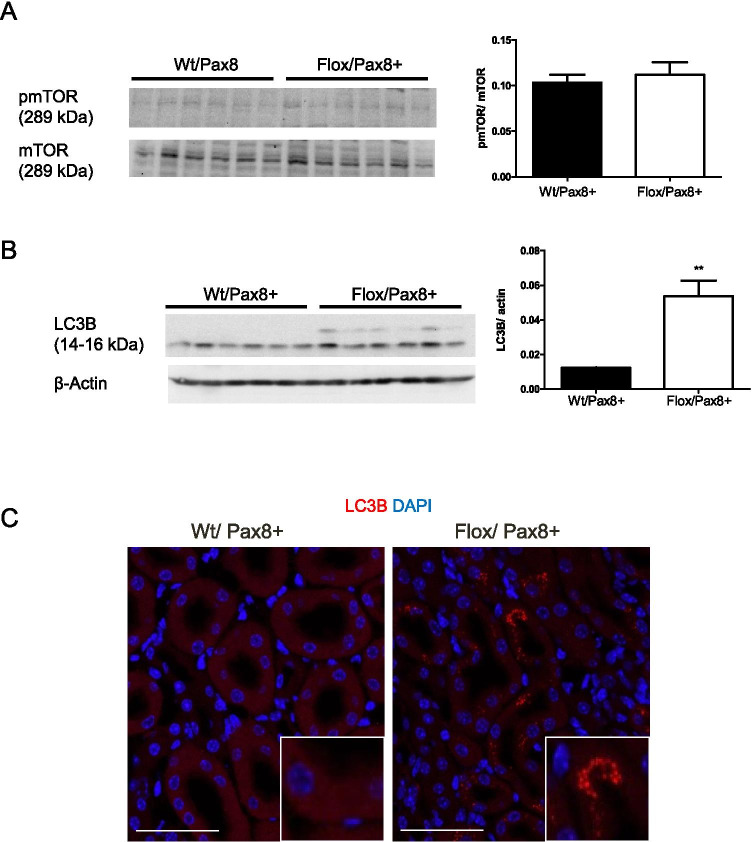
Accumulation of autophagosomes in ATP6ap2 deficient mice. **A**, **B** Western blotting for total and phosphorylated mammalian target of rapamycin (mTOR) and the LC3B subunit of the autophagosome and densitometry summarizing results. Student’s *t*-test (n = 4 per group), **p ≤ 0.01. **C** Immunohistochemistry for microtubule-associated proteins 1A/1B light chain 3B (LC3-B) (red) and DAPI (blue) in kidneys from Wt and Flox/Pax8 + mice insert shows higher magnification. Scale size bar 100 µm

mTOR signaling is linked to lysosomal function [5, 34, 74]. Vice versa, inhibition of mTOR by rapamycin in mice caused megalin downregulation and proteinuria through a H^+^-ATPase-dependent mechanism [21]. However, in Flox/Pax8 + kidneys, phosphorylated mTOR and total mTOR amount were unaltered (Fig. 8).

mTOR signaling and ATP6ap2 are linked to autophagy [29, 43, 59]. Immunoblotting and staining for the microtubule-associated protein 1A/1B-light chain 3 or LC3-B, a subunit of the autophagosome showed accumulation of LC3-B in proximal tubules from ATP6ap2 deficient mice but not in wild type controls (Fig. 8B, C).

## Discussion

In this study, we demonstrated that ATP6ap2 plays a role in receptor-mediated endocytosis and lysosomal function in the proximal tubule. Both *ATP6ap2-*Sh rats and Flox/Pax8Cre + mice showed excretion of albumin and low molecular weight proteins in urine, delayed processing of substrates of the receptor-mediated endocytosis pathway, and reduced expression of other key molecules involved in this process as well as accumulation of the LC3-B subunit of the autophagosome. All these changes are consistent with impaired lysosome function and reduced activity of the receptor-mediated endocytosis pathway.

We used two rodent models to demonstrate a role of ATP6ap2 in proximal tubule endocytosis of low molecular weight proteins and lysosomal function. Both models yield similar results as discussed below in more detail. The mouse model used is similar to previously published models with a more severe renal defect including an urinary concentration defect, salt loss, and distal renal tubular acidosis [56, 57, 70]. However, there are major differences between these models and our model. We induced Pax8-driven Cre activity in animals 6–8 weeks old whereas in the published models doxycycline was given already to mothers or to animals after weaning. Moreover, we used in the first model a lower doxycycline dose [44] to induce Cre activity and deletion of ATP6ap2 as we had previously observed that higher doses resulted also in a transient renal phenotype in animals not expressing any transgene. The low-dose doxycycline protocol resulted in an almost complete deletion of ATP6ap2 in the proximal tubule but with well-preserved expression in the collecting duct thereby circumventing a more severe renal damage and phenotype allowing to focus on proximal tubule functions. Likewise, the higher dose of doxycycline deleted ATPap2 from the proximal tubule but maintained most of the intercalated cell expression as evident from immunohistochemistry and normal urine pH. Consequently, our model did not develop signs of renal acidosis. This is important to consider as metabolic acidosis has recently been shown to impair proximal tubule endocytosis [11] and the defect described here is likely not caused by systemic alterations in acid–base balance.

ATP6ap2 associates with H^+^-ATPases [15, 36, 41, 52, 61]. We and others had shown that the ATP6ap2 is expressed in the renal proximal tubule and colocalizes with H^+^-ATPases in this nephron segments [17, 28, 60]. H^+^-ATPases contribute to several functions of the proximal tubule such as reabsorption of bicarbonate, trafficking and recycling of membrane proteins, or endocytosis of proteins from urine, acidification of vesicles along the endocytic pathway, and lysosomes processing or degrading absorbed substrates [71].

Several lines of evidence suggest that the ATP6ap2 modulates H^+^-ATPase function in the proximal tubule and thereby contributes to receptor-mediated endocytosis and lysosomal function. However, our data do not support a role of the ATP6ap2 in fluid-phase mediated endocytosis as suggested by data from Drosophila [25] or other generalized functions of the proximal tubule. Urine analysis and immunoblotting showed no generalized Fanconi-syndrome like loss of function as indicated by the absence of glucosuria, massive proteinuria, or changes in urine and blood pH. Both, Sh rats with reduced ATP6ap2 and ATP6ap2 KO mice, showed albuminuria in combination with the presence of low molecular weight proteins such as the vitamin D binding protein (VDBP) or procathepsin B. Also, injected recombinant transferrin was found in urine of Sh rats. Thus, these findings are indicative of reduced proximal tubular protein absorption. In the case of the Sh rats, we cannot exclude also increased filtration of albumin or transferrin as in these animals ATP6ap2 expression in podocytes may be reduced and podocyte-specific deletion of ATP6ap2 causes massive proteinuria and glomerular damage [49, 59]. Therefore, we created a mouse model with deletion of the ATP6ap2 in the epithelial cells along the nephron with intact expression of the protein in podocytes and found similar urinary excretion of albumin, VDBP, and procathepsin. Absorption of these proteins depends on the initial recognition by and binding to the multi-specific receptors megalin and cubilin present in the brush border membrane [47]. Deletion of the ATP6ap2 did not alter overall abundance of these receptors and also did not affect their localization at the level of light microscopy. Proteins bound to these receptors are then internalized in a receptor-ligand complex and further processed through early endosomes to late endosomes and lysosomes. In our experiments, four different ligands of these receptors were further investigated: albumin and VDBP which are internalized by both megalin and cubilin, procathepsin B which is preferentially absorbed by megalin, and transferrin a preferred substrate for cubilin [47]. All four proteins were found at elevated levels in urine suggesting a defect common to both receptors. The number of urine samples available for analysis was low precluding a more detailed analysis and more robust statistics. Nevertheless, more detailed analysis of transferrin internalization showed that transferrin is taken up from urine, albeit at somewhat lower rates (as indicated by higher urinary excretion after injection of transferrin) and that processing of transferrin to lysosomes is delayed as suggested by accumulation of transferrin 40 min after injection and much reduced colocalization of injected transferrin with a lysosomal marker (Lamp-1) at this later time point.

The delayed processing of endocytic substrates is most likely due to a reduced capacity of endocytic and lysosomal vesicles to acidify. A role of the ATP6ap2 in endosomal acidification has been described in Xenopus frog embryos and linked to reduced H^+^-ATPase activity [15]. Consistently, we found decreased expression of several H^+^-ATPase subunits including the a4 subunit (ATP6V0a4) in Sh rats and KO mice as well as the EII (ATP6V1EII) subunit in the KO mice. Similarly, ablation of the ATP6ap2 in embryonic cardiomyocytes reduced expression of several subunits of the V_0_ domain of the H^+^-ATPase and impaired lysosomal acidification [30]. In mice, deletion of ATP6V0a4 causes proximal tubular dysfunction and reduced receptor-mediated endocytosis with impaired lysosomal function consistent with an important role of H^+^-ATPases in these processes [22]. Whether the lower expression of the a4 subunit in the ATP6ap2 deficient rodent models is the consequence of absent ATP6ap2 function and/or directly causes the endocytic and lysosomal defect remains unclear at this point.

Defects in lysosomal acidification due to reduced or absent H^+^-ATPase function can cause altered mTOR signaling and activation of the autophagosome as in X-linked autophagic myopathy due to mutations in the VMA21 protein required for lysosomal H^+^-ATPase assembly [55] or in podocytes with specific deletion of the ATP6ap2 and impaired lysosomal function [49, 59]. Also in liver cells or pancreatic beta cells, absence or mutation of ATP6ap2 causes a lysosomal defect with impaired autophagy and vacuolation [7, 12, 62]. Fusion of lysosomes and autophagosomes occurs independently from lysosomal acidification but requires H^+^-ATPases [38]. Lysosomes act as signaling platforms associating with the mTOR complex 1 and H^+^-ATPase function is required for mTORC1 signaling whereas in turn mTOR signaling regulates H^+^-ATPase expression [50, 75]. mTOR is also a regulator of autophagosome function and autophagosome-lysosomal fusion and reduced mTOR signaling impairs autophagosome-lysosome fusion [27, 39]. Thus, deletion of the ATP6ap2 may impair H^+^-ATPase assembly and function, thereby decreasing endosomal and lysosomal acidification as well as mTOR signaling. Consequently, lysosome-autophagosome fusion may be decreased due to H^+^-ATPase-dependent but acidification-independent as well as mTOR-dependent mechanisms. Of note, increased levels of the ATP6ap2 in a podocyte cell line enhance mTOR signaling and reduce autophagosome expression and activity [35]. We found in the ATPap2 KO mouse models increased accumulation of the LC3B autophagosome subunit in proximal tubule cells whereas mTOR signaling appeared not to be affected.

In the rat model, Sh rats showed altered tubular morphology in H&E stained sections with vacuoles prominently present in proximal tubules. While the cause of these vacuoles remains elusive, the vacuoles potentially impair overall proximal tubule function and may particularly impact on endocytosis and vesicular trafficking contributing to the observed defect in endocytosis. However, the fact that a similar defect in endocytosis was also observed in the mouse models with apparently normal proximal tubular morphology (at least by light microscopy) strongly suggests that the loss of ATP6ap2 through mechanisms discussed above impairs endocytosis and processing of cargo.

In summary, genetic deletion of the (P)RR/ATP6ap2 from the proximal tubule in rats and mice causes albuminuria and low molecular weight proteinuria paralleled by reduced expression of several H^+^-ATPase subunits and delayed processing of substrates of receptor-mediated endocytosis to lysosomes. Lysosomal dysfunction is suggested by accumulation of the LC3-B subunit of the autophagosome. Thus, the ATPap2 facilitates receptor-mediated endocytosis in the proximal tubule possibly by modulating H^+^-ATPases.

## Supplementary Information

Below is the link to the electronic supplementary material.

Supplementary file1 (PDF 4.54 MB)

Supplementary file2 (DOCX 21.6 KB)
