# Supplementary material for: The (pro)renin receptor (ATP6ap2) facilitates receptor-mediated endocytosis and lysosomal function in the renal proximal tubule
Source: Pflugers Arch. 2021 Jul 6;473(8):1229–46. doi: 10.1007/s00424-021-02598-z (PMC8302575; doi:10.1007/s00424-021-02598-z)
Supplement: Supplementary file 1 — Supplementary file1 (PDF 4.54 MB) [file 424_2021_2598_MOESM1_ESM.pdf]

SUPPLEMENTARY FIGURE 1

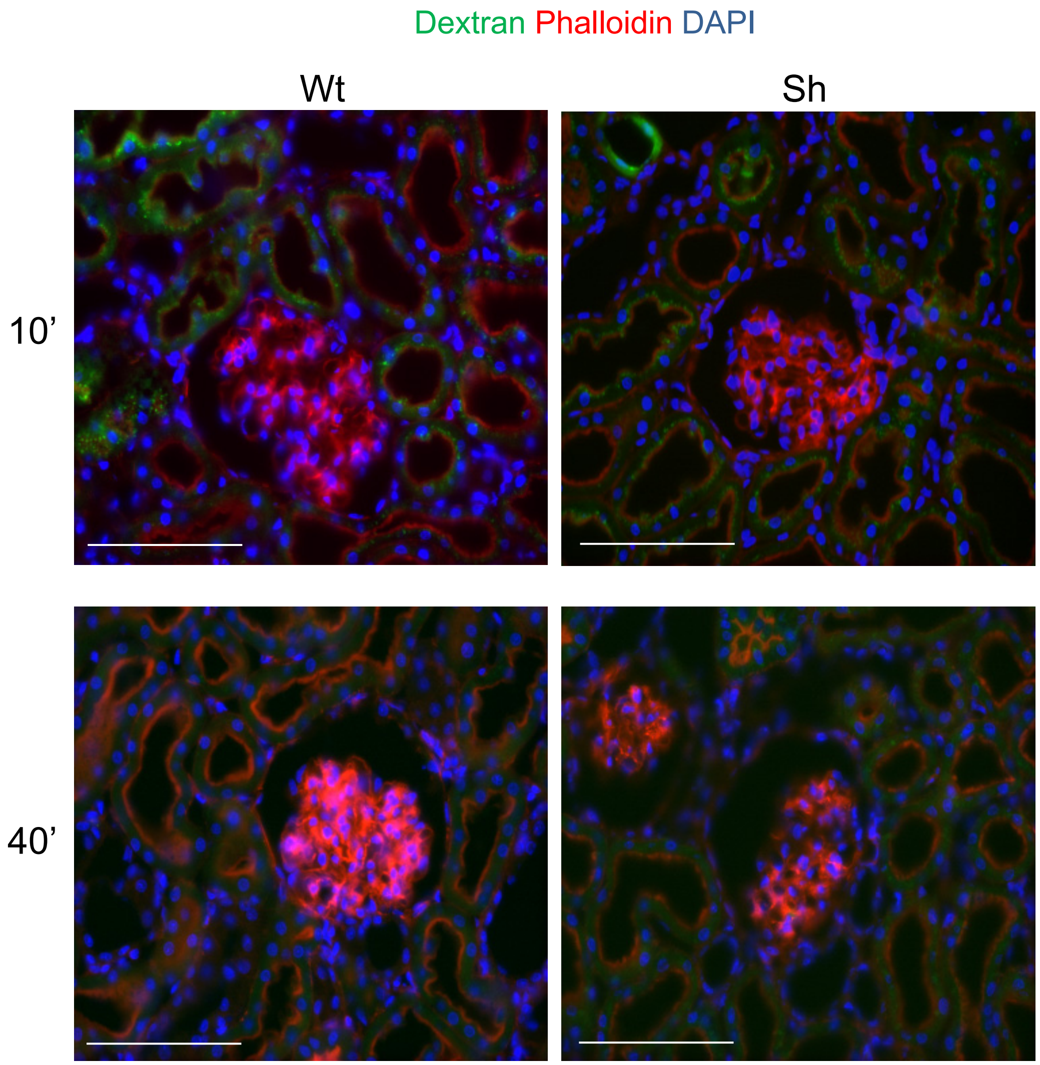

SUPPLEMENTARY FIGURE 2

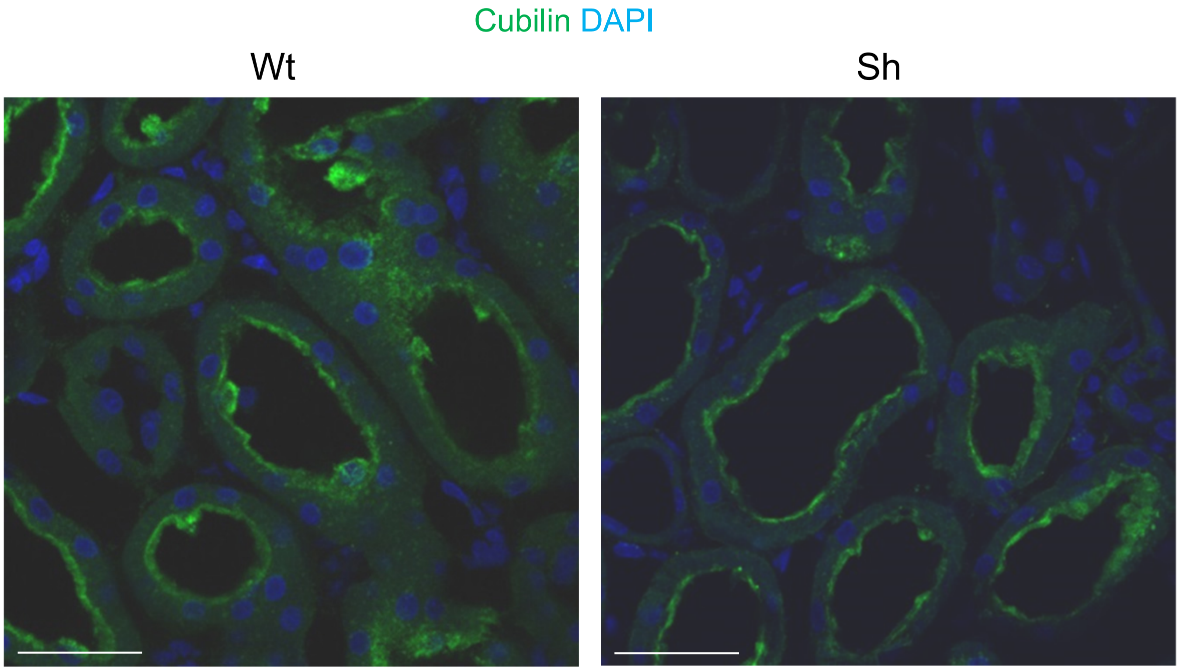

SUPPLEMENTARY FIGURE 3

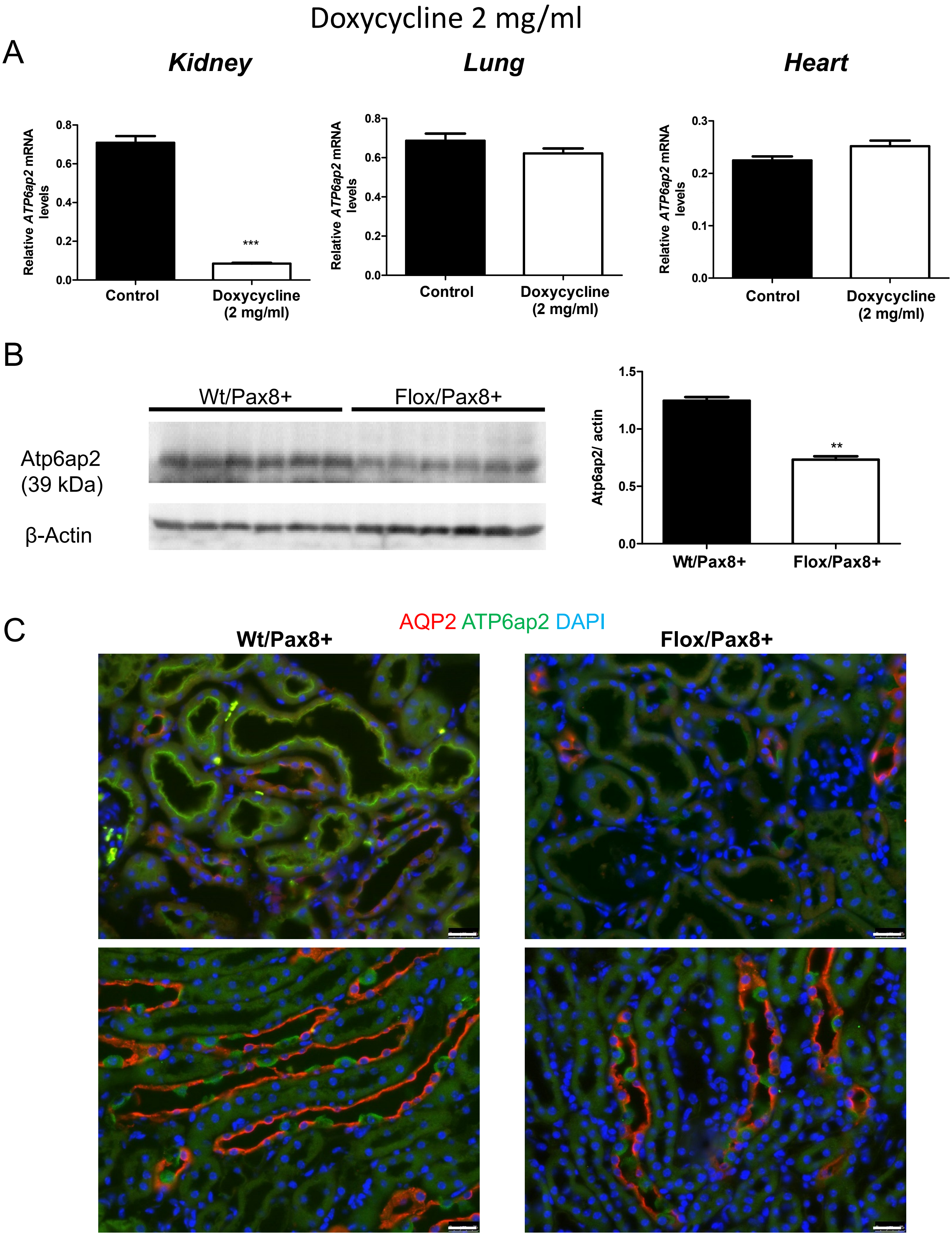

SUPPLEMENTARY FIGURE 3

Doxycycline 2 mg/ml

D

Wt/Pax8+ **Flox/Pax8+**

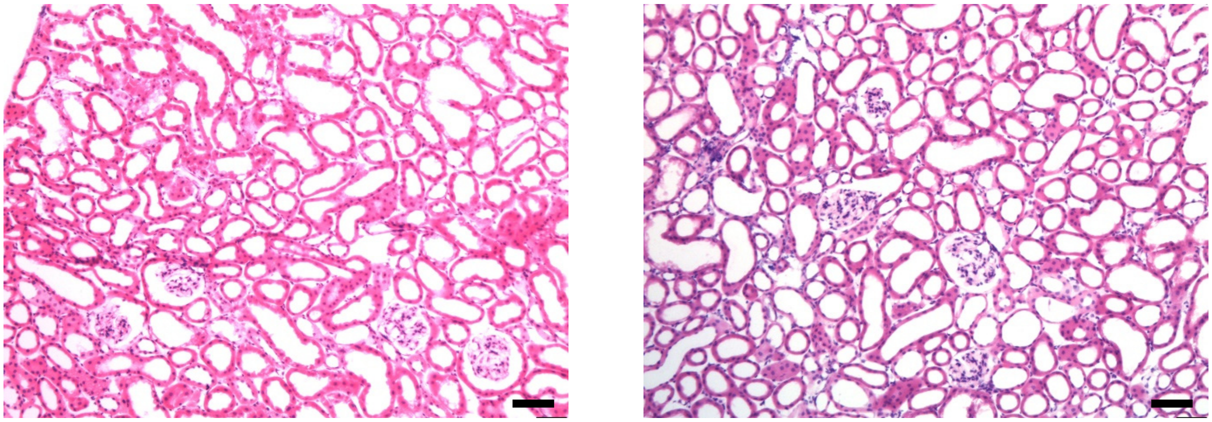

SUPPLEMENTARY FIGURE 4

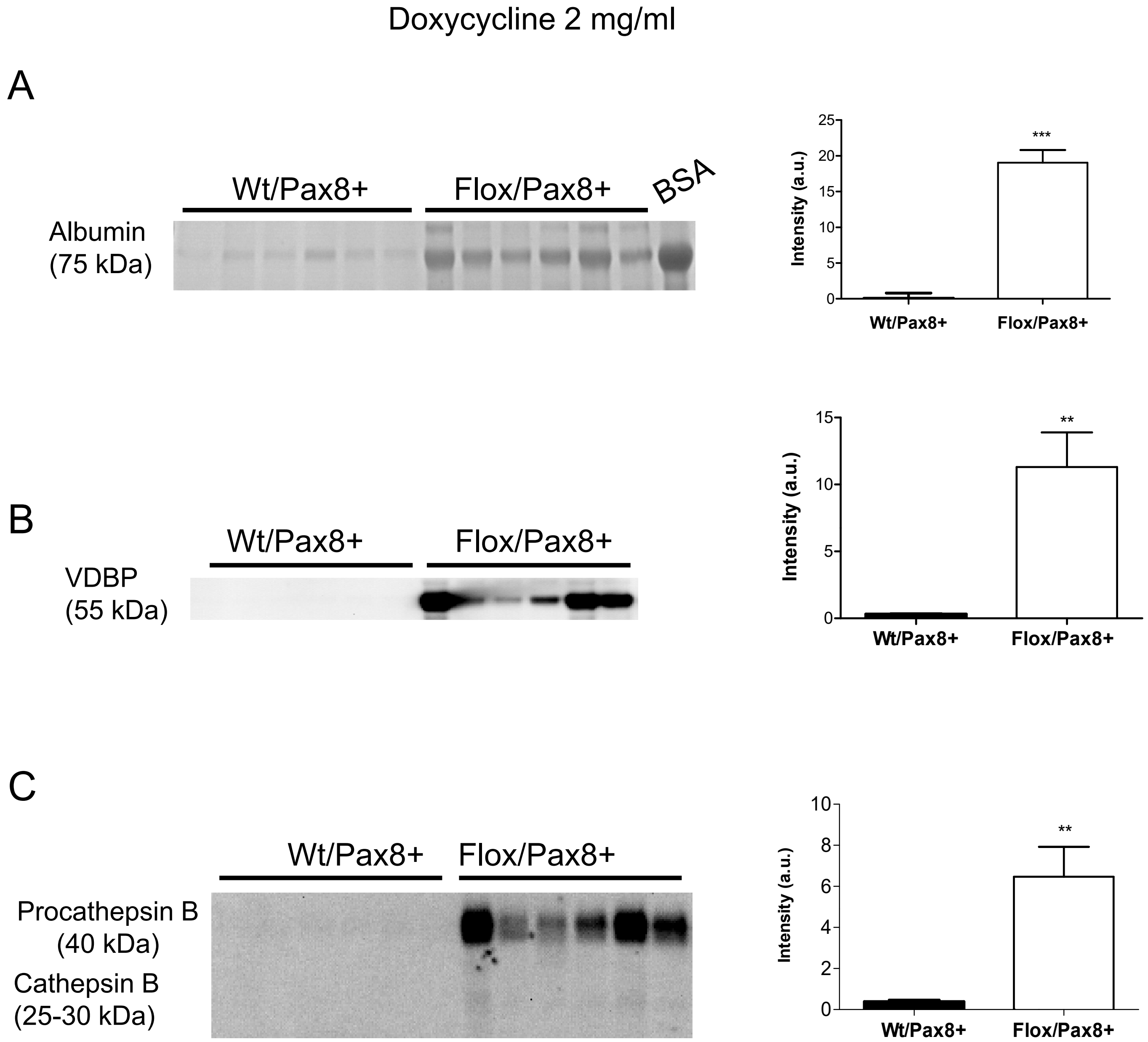

SUPPLEMENTARY FIGURE 5

Doxycycline 2 mg/ml

Lamp-1 Transferrin DAPI

Wt/Pax8+

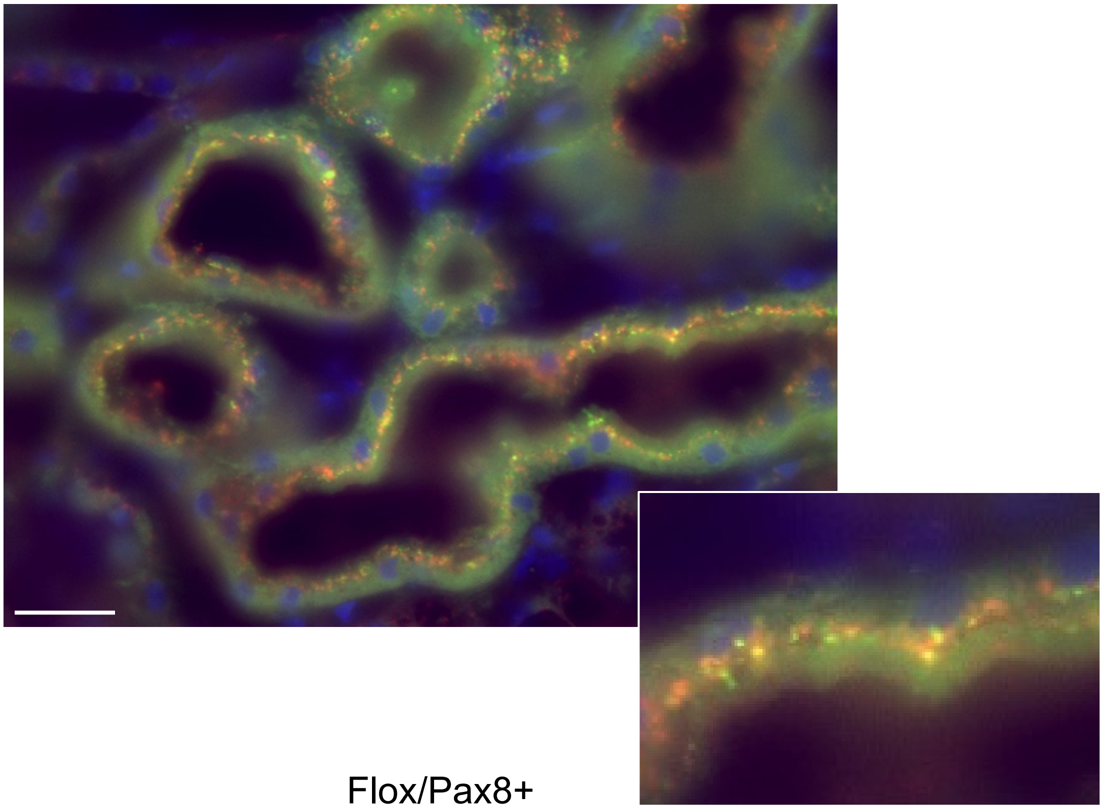

Flox/Pax8+

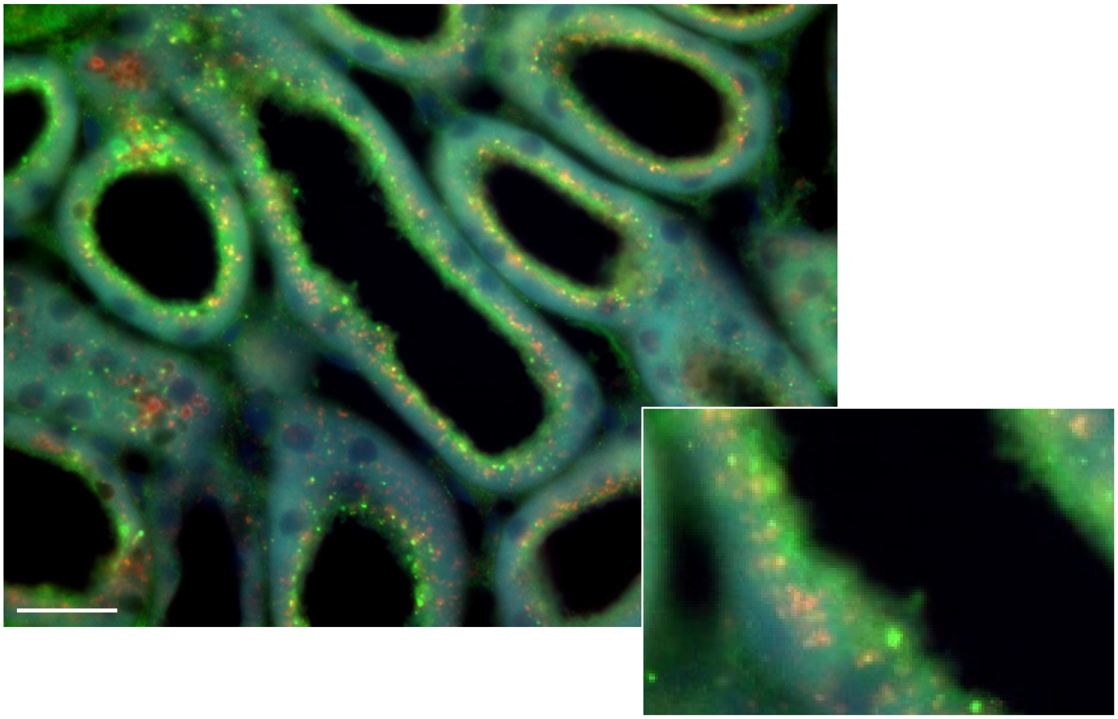

SUPPLEMENTARY FIGURE 6

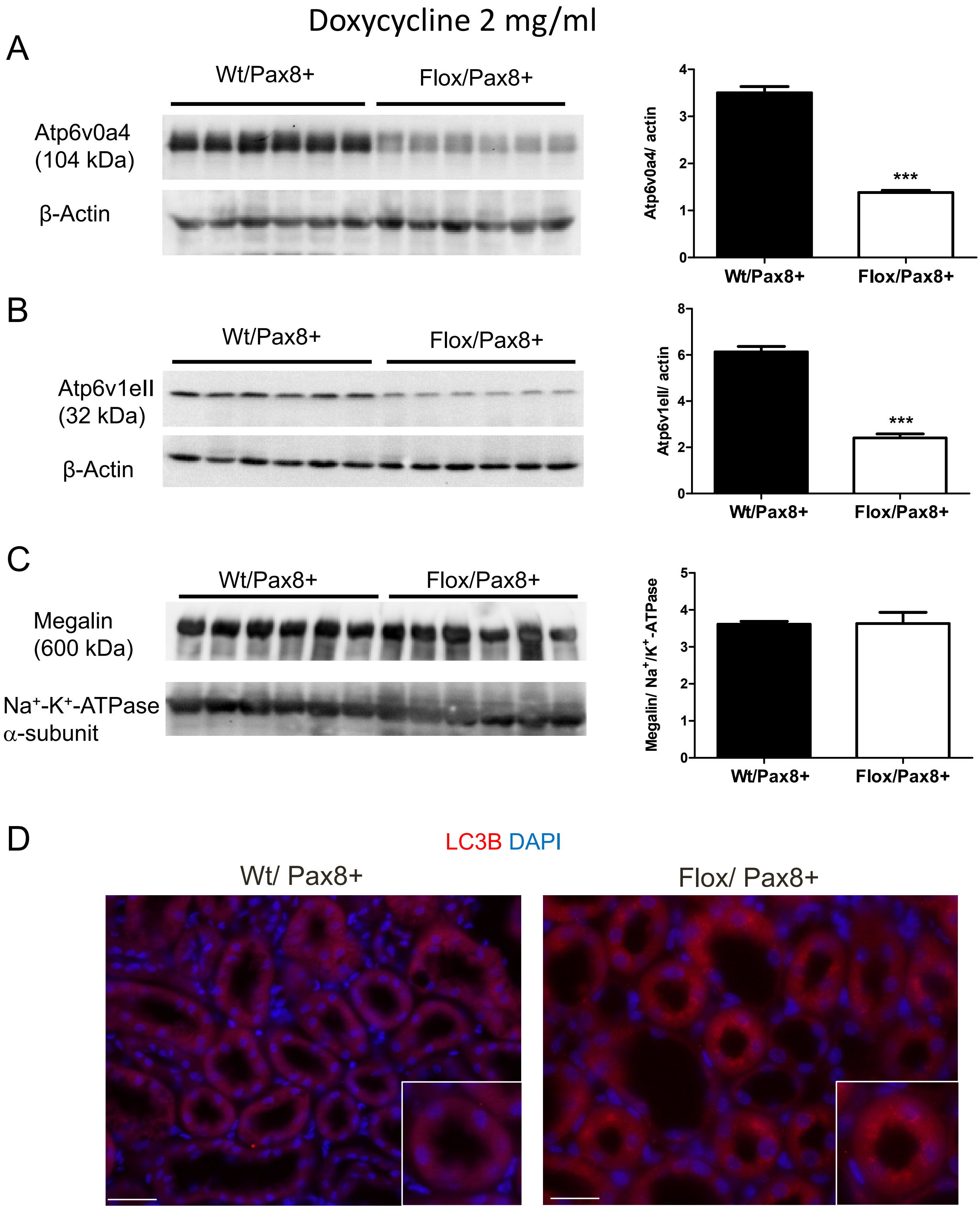

SUPPLEMENTARY FIGURE 7

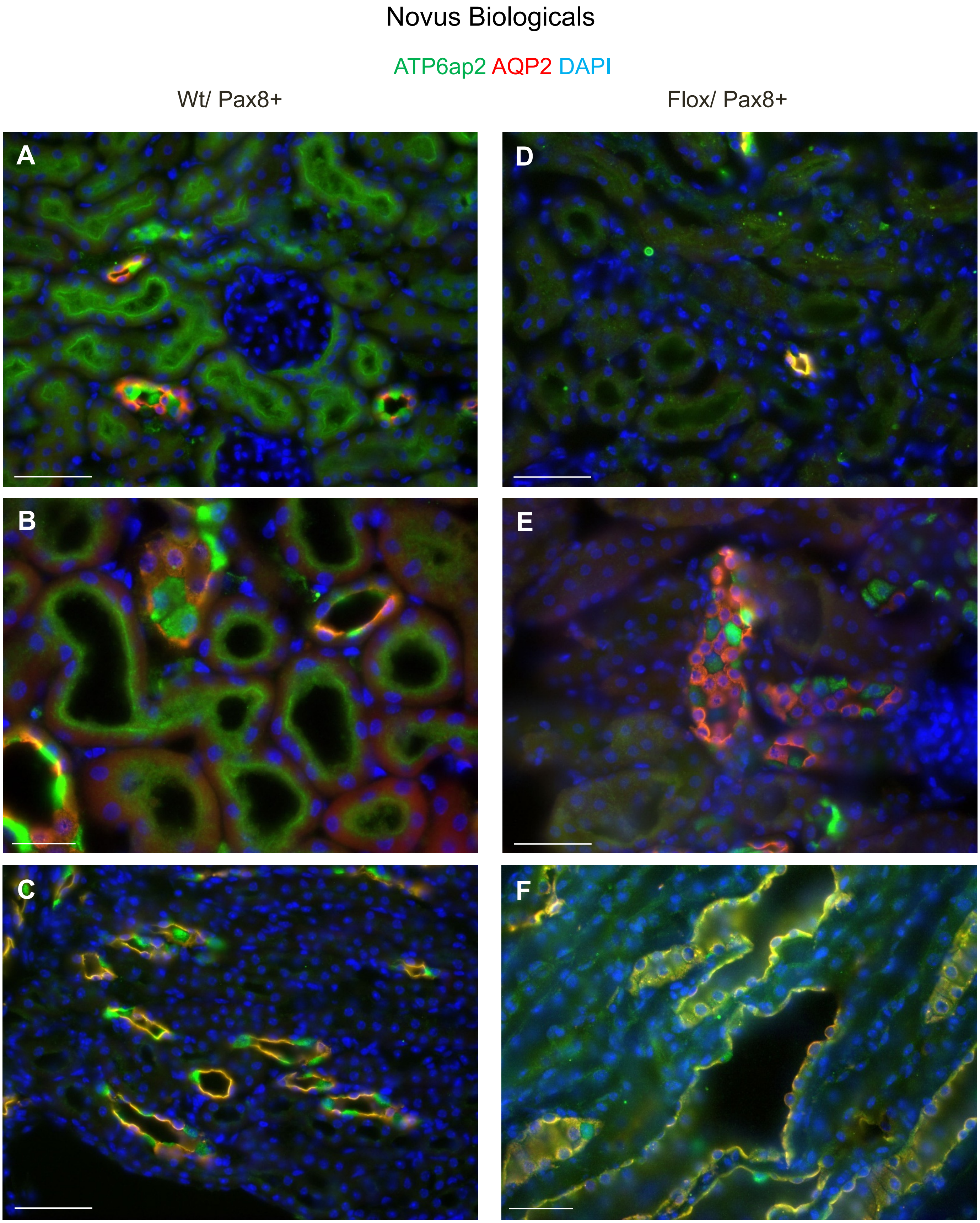

SUPPLEMENTARY FIGURE 8

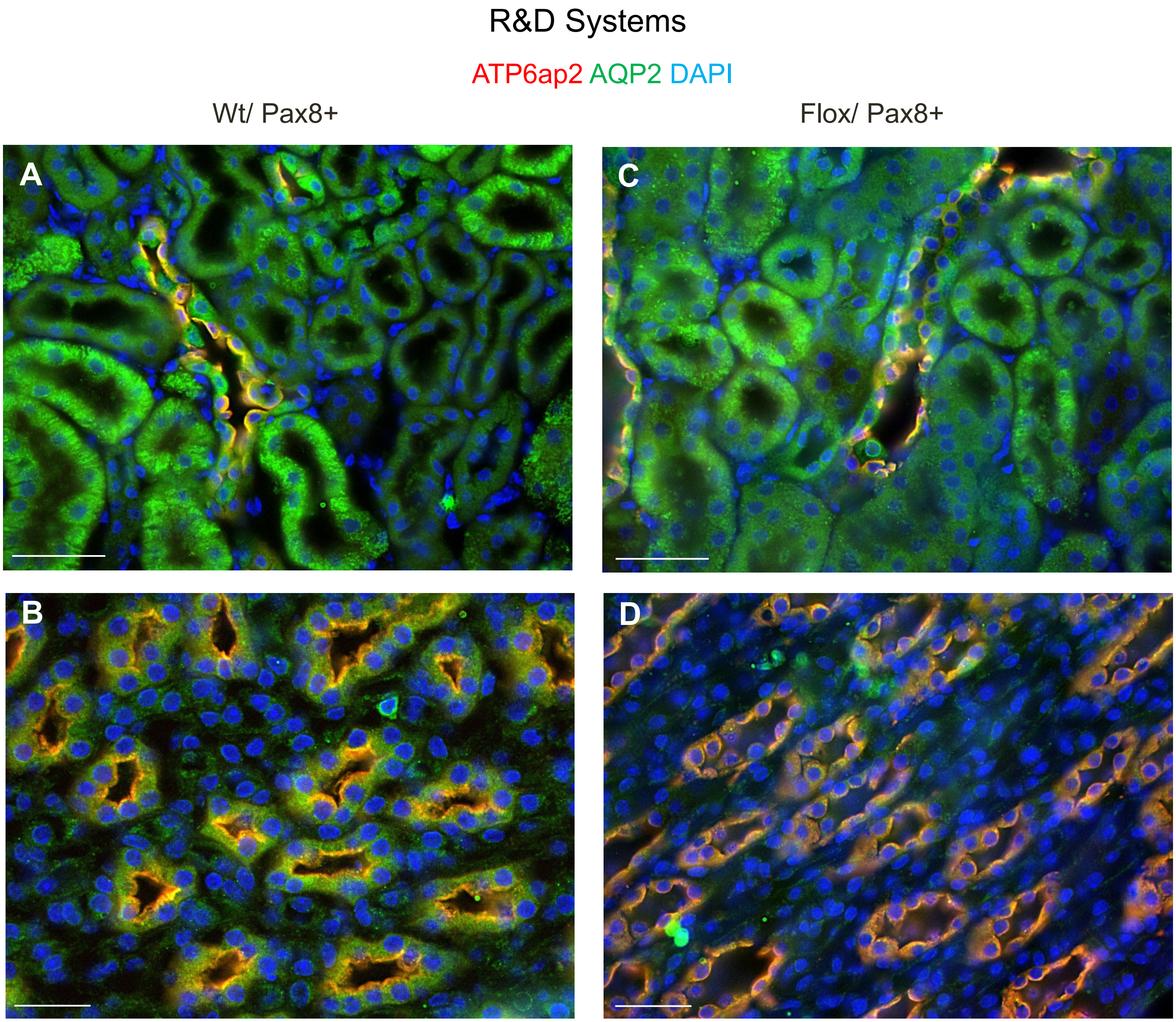

Supplementary figure 1. Dextran-FITC staining intensity and localization is similar in WT and shRNA rats at 10 min and 40 min time points.

Immunohistochemistry for dextran-FITC, 10 kDa (green), DAPI (blue), and actin/phalloidin (red) in kidney slices from Wt and shRNA rats 10 and 40 min after injection. Scale size bar 100 μm.

Supplementary figure 2. Cubilin localization in control and shRNA rat kidneys.

Immunohistochemistry for cubilin (green) and DAPI (blue) in kidney slices from Wt and shRNArats. Scale size bar 50 μm.

Supplementary figure 3. Generation of kidney epithelial cell specific ATP6ap2 ablation in mouse using the higher doxycycline protocol.

Mice received 2 mg/ml doxycycline as described in the methods section and ablation of *ATPap2* in kidney was examined. **(A)** RT-qPCR analysis of total mRNA of kidneys, lungs and hearts from Wt and Flox/Pax8+ mice. *Atp6ap2* mRNA abundance was normalized to *HPRT*. Statistical analysis was performed using Student's *t*-test (Wt/Pax8+: n = 6 and Flox/Pax8+: n = 6) *** $p < 0.001$. **(B)** Western blotting for ATP6ap2 in total membrane preparations from kidney of Wt and Flox/Pax8+ mice and summary of data (normalized to β-actin-loading) as bar graph. Statistical analysis was performed using Student's *t*-test (Wt/Pax8+: n = 6 and Flox/Pax8+: n = 6) ** $p < 0.01$. **(C)** Immunohistochemistry for ATP6ap2 (green), AQP2 (red), and DAPI (blue) in kidney sections from Wt/Pax8 and Flox/Pax8+ mice. Scale size bar 25 μm. **(D)** H&E stained kidney sections from Wt/Pax8 and Flox/Pax8+ mice. Scale size bar 25 μm.

Supplementary figure 4. Albuminuria and low molecular weight proteinuria in the absence of the ATP6ap2

Animals were treated with the higher dose of doxycycline (2 mg/ml). 24 hrs urine samples were normalized to creatinine. Bovine serum albumin (BSA, 7 mg/ml) was loaded as positive control. **(A)** Albumin was detected by coomassie blue staining whereas **(B)** vitamin D binding protein (VDBP) or **(C)** (Pro)cathepsin B were revealed by immunoblotting. Data were summarized as bar graphs (n = 6/genotype. Student's *t*-test ** $p < 0.01$, *** $p < 0.001$.

Supplementary figure 5. Delayed receptor-mediated endocytosis in ATP6ap2 deleted mice

WT/Pax8+ and Flox/Pax8+ mice were pretreated with 2 mg/ml doxycycline. Mice were injected with

human recombinant transferrin and kidneys collected 40 min after injection. Immunohistochemistry for human transferrin (green), DAPI (blue), and the lysosomal protein Lamp-1 (red) was performed. Inserts show higher magnifications. Scale size bar 25 μm.

Supplementary figure 6. Deletion of the ATP6ap2 alters expression of H^+-ATPase subunits and autophagosome subunit

Mice were treated with the higher dose of doxycycline (2 mg/ml). (**A-C**) Total membrane preparations from kidneys of Wt/Pax8+ and Flox/Pax8+ mice were blotted for the H^+ATPase a4 (ATP6V0a4) and EII (ATP6V1EII) subunits as well as for megalin and the alpha subunit of the Na^+/K^+-ATPase as loading control. Densitometries were normalized to β-Actin or Na^+/K^+-ATPase (loading control). Student`s *t*-test (n = 6 per group), *** $p \leq 0.001$ **(D)** Immunohistochemistry for the Microtubule-associated proteins 1A/1B light chain 3B (LC3-B) (red) and DAPI (blue) in kidneys from Wt and Flox/Pax8+ mice, insert shows higher magnification. Scale size bar 25 μm.

Supplementary figure 7. Detection of ATP6ap2 in mouse kidneys with various antibodies

Kidneys from wildtype (Wt/ Pax8+)**(A-C)** and Flox/Pax8+ **(D-F)** mice were stained with antibodies from Novus Biologicals against the (P)RR/ATP6ap2 (green), AQP2 (red), and with DAPI (blue) to detect nuclei. In wildtype kidney, strong staining with antibodies against the (P)RR/ATP6ap2 was detected in proximal tubules and in collecting ducts, in Flox/Pax8+ mouse kidney, the staining was weaker in the proximal tubule but preserved in collecting ducts. Of note, all cell types in the collecting duct were stained. Scale size bar: A,C,D,E: 100 μm, B,F: 50 μm.

Supplementary figure 8. Detection of (P)RR/ATP6ap2 in mouse kidneys with various antibodies

Kidneys from wildtype (Wt/ Pax8+)**(A-B)** and Flox/Pax8+ **(C-D)** mice were stained with antibodies from R&D Systems against the (P)RR/ATP6ap2 (green), AQP2 (red), and with DAPI (blue) to detect nuclei. In wildtype kidney, strong staining with antibodies against the (P)RR/ATP6ap2 was detected in proximal tubules and in collecting ducts while in the Flox/Pax8+ mouse kidneys, staining in the proximal tubule and collecting ducts remained unchanged. In contrast to staining with other antibodies, staining with R&D Systems antibodies was mostly intracellular. Scale size bar: A,C: 100 μm, B,D: 50 μm.
