# Supplementary material for: The (pro)renin receptor (ATP6ap2) facilitates receptor-mediated endocytosis and lysosomal function in the renal proximal tubule
Source: Pflugers Arch. 2021 Jul 6;473(8):1229–46. doi: 10.1007/s00424-021-02598-z (PMC8302575; doi:10.1007/s00424-021-02598-z)
Supplement: Supplementary file 2 — Supplementary file2 (DOCX 21.6 KB) [file 424_2021_2598_MOESM2_ESM.docx]

**Supplementary Table 1: higher dose doxycycline (2 mg/ml)**

| **Parameters** | **Wt/Pax81** | **Flox/Pax81** |
| --- | --- | --- |
| Body weight (g) | 31.2 ± 0.7 | 29.2 ± 0.8 |
|  |  |  |
| ***Plasma*** |  |  |
| Na^+^ (mM) | 147.7 ± 1.5 | 147.2 ± 0.5 |
| K^+^ (mM) | 5.0 ± 0.1 | 4.8 ± 0.1 |
| Cl^-^ (mM) | 113.0 ± 1.7 | 112.0 ± 0.6 |
| Prealbumin (mg/dL) | 0.80 ± 0.10 | 1.42 ± 0.40 |
| Creatinine (mg/dL) | 0.06 ± 0.01 | 0.14 ± 0.01 ** |
| BUN (mg/dL) | 41.7 ± 1.6 | 46.5 ± 3.2 |
|  |  |  |
| **Urine** | | |
| Urine (g/g BW) | 0.15 ± 0.01 | 0.18 ± 0.01 |
| Creatinine (mg/dL) | 78.6 ± 10.1 | 58.6 ± 10.3 |
| Creatinine clearance (mL/min) | 4.04 ± 0.47 | 1.57 ± 0.41 * |
| Urine pH | 6.40 ± 006 | 6.50 ± 0.07 ** |
